# Polyribosome-Dependent Clustering of Membrane-Anchored RNA Degradosomes To Form Sites of mRNA Degradation in Escherichia coli

**DOI:** 10.1128/mBio.01932-21

**Published:** 2021-09-07

**Authors:** Lina Hamouche, Leonora Poljak, Agamemnon J. Carpousis

**Affiliations:** a LMGM, Université de Toulouse, CNRS, UPS, CBI, Toulouse, France; b TBI, Université de Toulouse, CNRS, INRAE, INSA, Toulouse, France; National Cancer Institute

**Keywords:** RNA degradosome, mRNA degradation, inner cytoplasmic membrane, polyribosome, rifampin, kasugamycin, chloramphenicol

## Abstract

The essential endoribonuclease RNase E, which is a component of the Escherichia coli multienzyme RNA degradosome, has a global role in RNA processing and degradation. RNase E localizes to the inner cytoplasmic membrane in small, short-lived clusters (puncta). Rifampin, which arrests transcription, inhibits RNase E clustering and increases its rate of diffusion. Here, we show that inhibition of clustering is due to the arrest of transcription using a rifampin-resistant control strain. Two components of the RNA degradosome, the 3′ exoribonuclease polynucleotide phosphorylase (PNPase) and the DEAD box RNA helicase RhlB, colocalize with RNase E in puncta. Clustering of PNPase and RhlB is inhibited by rifampin, and their diffusion rates increase, as evidenced by *in vivo* photobleaching measurements. Results with rifampin treatment reported here show that RNA degradosome diffusion is constrained by interaction with RNA substrate. Kasugamycin, which arrests translation initiation, inhibits formation of puncta and increases RNA degradosome diffusion rates. Since kasugamycin treatment results in continued synthesis and turnover of ribosome-free mRNA but inhibits polyribosome formation, RNA degradosome clustering is therefore polyribosome dependent. Chloramphenicol, which arrests translation elongation, results in formation of large clusters (foci) of RNA degradosomes that are distinct from puncta. Since chloramphenicol-treated ribosomes are stable, the formation of RNA degradosome foci could be part of a stress response that protects inactive polyribosomes from degradation. Our results strongly suggest that puncta are sites where translationally active polyribosomes are captured by membrane-associated RNA degradosomes. These sites could be part of a scanning process that is an initial step in mRNA degradation.

## INTRODUCTION

RNase E is an essential endoribonuclease with major roles in maturation of stable RNA, degradation of mRNA, and posttranscriptional regulation of gene expression (1–6). Instability of mRNA is important for regulation of gene expression because it permits rapid remodeling of the transcriptome. Degradation involves fragmentation of mRNA by endoribonucleases, principally RNase E, followed by digestion to nucleotides by exoribonucleases and oligoribonuclease (5, 7, 8). tRNA maturation and mRNA degradation are essential functions of RNase E (9–13).

The quaternary structure of RNase E is a homotetramer of 118-kDa monomers. The N-terminal half of the monomer folds into subunits that associate to form the catalytic core of RNase E (14, 15). The C-terminal half is a large noncatalytic region, which is mostly natively unstructured protein (16). Embedded within the noncatalytic region are small linear motifs (SLiMs), also known as microdomains, which are sites of interaction with other macromolecules (4, 17, 18). The noncatalytic region includes sites for interaction with the DEAD box RNA helicase RhlB, the glycolytic enzyme enolase, and the 3′ exoribonuclease polynucleotide phosphorylase (PNPase) (19–23). The multienzyme complex of RNase E, RhlB, enolase, and PNPase is known as the RNA degradosome (24–27). Experimental evidence has shown that RhlB, RNase E, and PNPase act coordinately as components of the RNA degradosome in the processing and degradation of RNA (28, 29). Mutant strains of Escherichia coli in which the RNA degradosome has been disrupted by deletion of part or all of the RNase E noncatalytic region are viable (20, 30). Nevertheless, disruption of the RNA degradosome has been shown to reshape the transcriptome and proteome (31, 32) and to result in defects in processes including initiation of mRNA degradation, small RNA (sRNA)-mediated gene silencing, and turnover of intermediates in mRNA degradation and of hypomodified tRNA (28, 33–35).

Recent work has shown that RNase E, RhlB, and PNPase are localized to the inner cytoplasmic membrane of E. coli (36, 37). RNase E has a 15-residue SLiM, the membrane-targeting sequence (MTS), which is located about 50 residues from the catalytic domain and forms an amphipathic alpha helix upon insertion into the phospholipid bilayer (36–38). The localization of RhlB and PNPase to the inner membrane requires their association with RNase E (36, 37). Quantification of superresolution images of live cells shows that RNA degradosome components are highly enriched on the inner cytoplasmic membrane and that deletion of the MTS results in their localization to the cytoplasm (37). Although it has been suggested that the membrane localization of RNase E preferentially destabilizes mRNA encoding inner-membrane proteins (37), another study found a global slowdown in mRNA degradation when the MTS is deleted but no correlation between changes in stability and the function or cellular location of the encoded proteins (39). The latter study suggests that membrane association of the RNA degradosome has a wide-ranging role in mRNA turnover.

Imaging of live cells by epifluorescence and total internal reflection fluorescence microscopy (TIRFm) has shown that RNase E-yellow fluorescent protein (YFP) forms short-lived clusters on the inner cytoplasmic membrane (36). Although we previously called these clusters foci, we now use the term “puncta” to distinguish small clusters of RNase E in the inner membrane of E. coli from large foci formed by cytoplasmic RNase E in Caulobacter crescentus (40). Rifampin treatment, which inhibits transcription (41, 42), disperses RNase E-YFP puncta. Within a few minutes of treatment, mRNA and precursors of tRNA and rRNA are depleted, resulting in arrest of protein synthesis and inhibition of growth. This arrest is accompanied by the degradation of 23S and 16S rRNA, a brief eclipse in cell viability, and a 50% decrease in cell size due to a terminal cell division (43). Ectopic expression of a hybrid *lacZ*-tRNA_Arg5_ transcript by bacteriophage T7 RNAP, which is not inhibited by rifampin, restored RNase E-YFP clustering in rifampin-treated cells (36). Photobleaching measurements showed that rifampin treatment relaxes constraints on the diffusion of RNase E-YFP. These results are evidence that the clustering of RNase E-YFP to form short-lived puncta is dependent on the presence of RNA substrate.

Here, we made strains with single-copy chromosomal constructs of RNase E, RhlB, and PNPase tagged with mCherry or monomeric superfolder green fluorescent protein (msfGFP), and we introduced a mutation that results in resistance to the drug rifampin. We show that RNase E, RhlB, and PNPase colocalize in puncta on the inner membrane of E. coli. Photobleaching measurements showed that rifampin treatment relaxes constraints on the rate of diffusion of RhlB and PNPase, as was previously shown for RNase E (36). We show that punctum formation and dynamics of RNase E, RhlB, and PNPase are unchanged upon drug treatment of rifampin-resistant strains. These results show that RNase E, RhlB, and PNPase act together as components of the RNA degradosome in clustering to form puncta on the inner cytoplasmic membrane of E. coli. Kasugamycin, which is a drug that inhibits the initiation of translation, has effects similar to those of rifampin. This result shows that the clustering of the RNA degradosome to form short-lived puncta depends on the presence of polyribosomes. Treatment with chloramphenicol, which inhibits translation elongation, results in large clusters (foci) of RNase E-mCherry that are distinct from the puncta observed in untreated cells. The formation of foci could stockpile RNA degradosomes as part of a mechanism that protects inactive polyribosomes from degradation. We discuss the implication of our results for the cell biology of mRNA degradation in Escherichia coli and related Gram-negative bacteria.

## RESULTS

We recently constructed and characterized an E. coli K-12 strain (SLM001) that is rifampin resistant (43). SLM001 has a mutation in the *rpoB* gene, which encodes the β-subunit of RNA polymerase. The isogenic *rpoB^+^* and *rpoB*(*D516Y*) strains Kti162 and SLM001 are E. coli K-12 derivatives that encode RNase E-mCherry at the *rne* locus under the control of endogenous expression signals (Table 1). The isogenic strain SLM001 grows on LA plates at temperatures ranging from 20 to 43°C with no visible difference in colony size or morphology compared to Kti162. In LB cultures at 37°C with vigorous shaking, Kti162 and SLM001 have the same growth rate (doubling time, 21 min).

**TABLE 1:** Strains and plasmids

Strain or plasmid	Genotype^*a*^	Reference
Strains		
NCM3416	E. coli K-12, F^−^ λ^−^ *rph^+^ zib-207*::Tn*10*	62
Kti162	NCM3416, *rne-mch*	35
Kti164	NCM3416, *rne-gfp*	35
SLM001	NCM3416, *rne-mch rpoB*(*D516Y*)	43
SLM018	NCM3416, *pnp-msfgfp*	This work.
SLM019	NCM3416, *rne-mch pnp-msfgfp*	This work.
SLM024	NCM3416, *rhlB-msfgfp*	This work.
SLM025	NCM3416, *rne-mch rhlB-msfgfp*	This work.
SLM027	NCM3416, *pnp-msfgfp rpoB*(*D516Y*)	This work.
SLM029	NCM3416, *rhlB-msfgfp rpoB*(*D516Y*)	This work.
SLM035	NCM3416, *atpB-msfgfp*::Kan	This work.
SLM036	NCM3416, *rne-mch atpB-msfgfp*::Kan	This work.
SLP122	NCM3416, pLP120	This work.
LHS590	NCM3416, *rne*Δ*MTS-FH*::*cat*	This work.
SAJ259	NCM3416, *rne*Δ*MTS-FH*::*cat pnp-msfgfp*	This work.
SAJ260	NCM3416, *rne*Δ*MTS-FH*::*cat rhlB-msfgfp*	This work.

Plasmids		
pSAB11	pAM238 derivative, *ori* pSC101 *spcR* Δ*lacOPZ*′	70
pLP120	pSAB11 derivative, *lacIOPZ*′*-rne*(*568–592*)*-msfgfp*	This work.

a *mch*, mCherry gene. The *atpB-msfGFP*::*kan* construct contains a kanamycin resistance cassette that is cotranscribed with *atpB-msfGFP*. The cassette cannot be removed by FLP recombinase, since there are no *frt* sites. This construct was designed to avoid interference with expression of *atp* genes downstream of *atpB*. The *rne*Δ*MTS-FH* construct encodes a 14-residue C-terminal FLAG epitope and 6-His sequence (DYKDDDDKHHHHHH). The *lacIOPZ*′*-rne*(*568–592*)*-msfgfp* sequence in plasmid pLP120 encodes the first 8 residues of LacZ followed by the MTS of RNase E and msfGFP; expression is under the control of *lacI* in its natural position upstream of *lacOP*.

### Inhibition of RNA degradosome clustering by rifampin.

**FIG 1:**
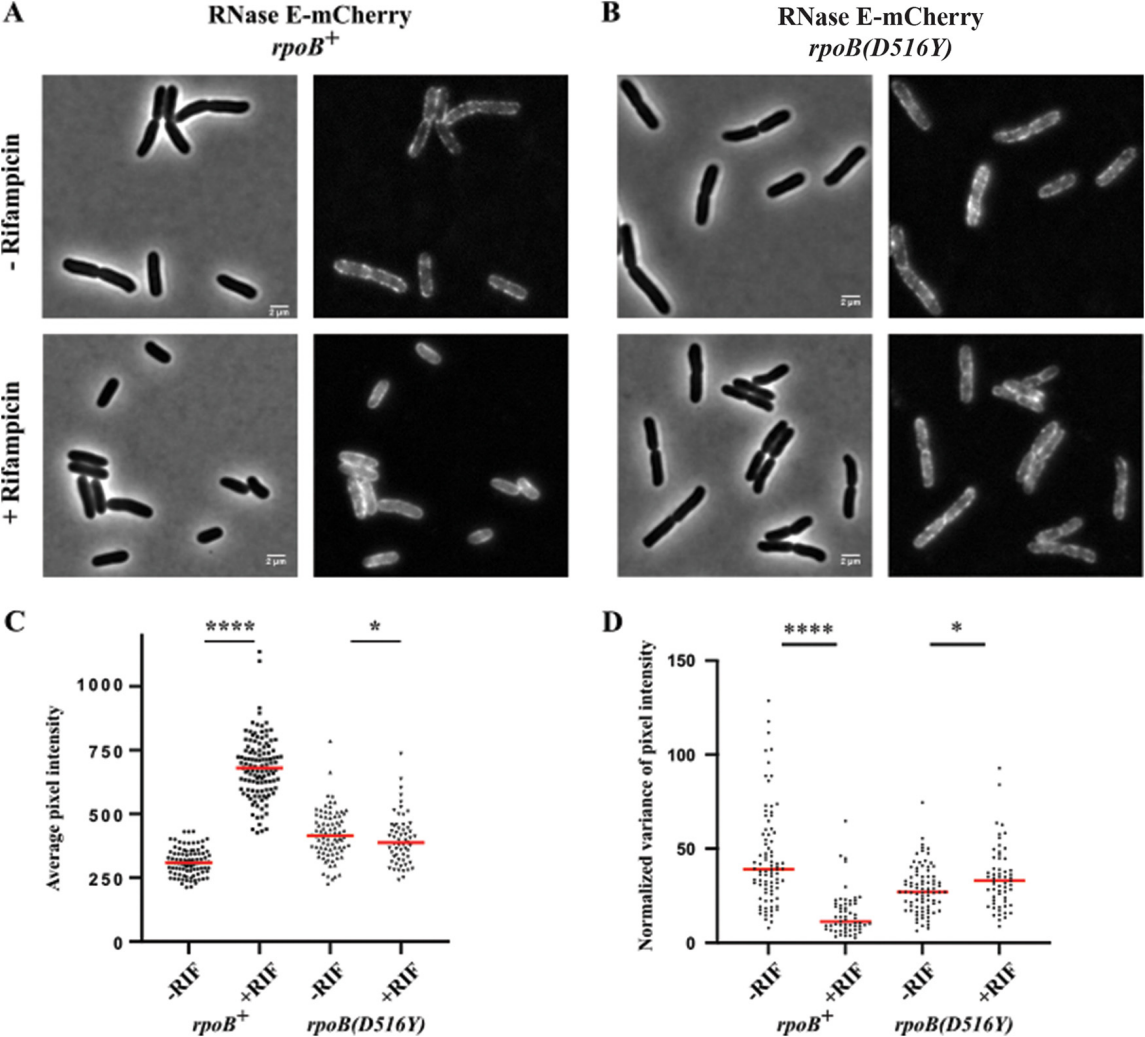
Inhibition of RNase E-mCherry punctum formation by rifampin. (A and B) A Kti162 strain (*rpoB^+^*) expressing RNase E-mCherry (A) and the isogenic SLM001 strain [*rpoB*(*D516Y*)] (B) were grown to an optical density at 600 nm (OD_600_) of 0.5 to 0.6 in LB at 37°C. Phase-contrast and epifluorescence images of RNase E-mCherry were captured before and after treatment with rifampin for 30 min. Strains with the *rpoB*(*D516Y*) mutation are resistant to inhibition by rifampin. Bar, 2 μm. Nonadjacent cells before and after treatment with rifampin were scanned along the periphery parallel to the long axis of the cell to measure average pixel intensity (C) and normalized variance in average pixel intensity (D). The graphs represent 60 to 100 line scans for each measurement. The red horizontal line in each plot marks the median. Statistical significance of the differences between untreated and rifampin-treated cells was calculated using the nonparametric Mann-Whitney test (****, *P < *0.0001; *, 0.01< *P* < 0.05).

mCherry is a monomeric red fluorescence protein that is well suited for pairing with green fluorescence protein (GFP) (44). Figure 1A shows the distribution of RNase E-mCherry in Kti162 (*rpoB^+^*) before and 30 min after rifampin addition. The puncta, which are conspicuously present (Fig. 1A, top), dispersed after rifampin treatment (bottom). We used line scans parallel to the long axis of the cell to measure the fluorescence intensity and the variance in average pixel intensity along the periphery of the cell (36, 45). Upon rifampin treatment, there is a large increase in average pixel intensity in Kti162 (*rpoB^+^*) (Fig. 1C). Since rifampin treatment arrests synthesis of new RNase E-mCherry, the increase in average pixel intensity is due to the maturation of mCherry. In E. coli, mCherry takes more than 1 h to mature (46). The 21-min cell doubling time, in contrast to more than 1 h for mCherry to mature, results in a large pool of immature mCherry that continues to mature after the arrest of mCherry synthesis.

The decrease in the normalized variance of average pixel intensity is a quantitative measure of the dispersion of RNase E-mCherry in a population of cells treated with rifampin (Fig. 1D). Inhibition of punctum formation by rifampin is comparable to previously described effects on RNase E-YFP (36). These results show that the clustering of RNase E-YFP on the inner cytoplasmic membrane is not an artifact due to ectopic expression from a plasmid or a tendency of YFP fusion proteins to aggregate (47).

Rifampin had no effect on the formation of RNase E-mCherry puncta in the SLM001 strain (Fig. 1B). Since there is only a small change in average pixel intensity, the proportion of mature mCherry molecules is nearly the same 30 min after the addition of rifampin due to growth in the presence of the antibiotic (Fig. 1C). Quantification of the normalized variance in average pixel intensity for a population of cells confirms that there is at most a small increase that could be due to growth after rifampin addition (Fig. 1D). These results are proof that the dispersion of RNase E clusters by rifampin treatment is due to inhibition of transcription.

**FIG 2:**
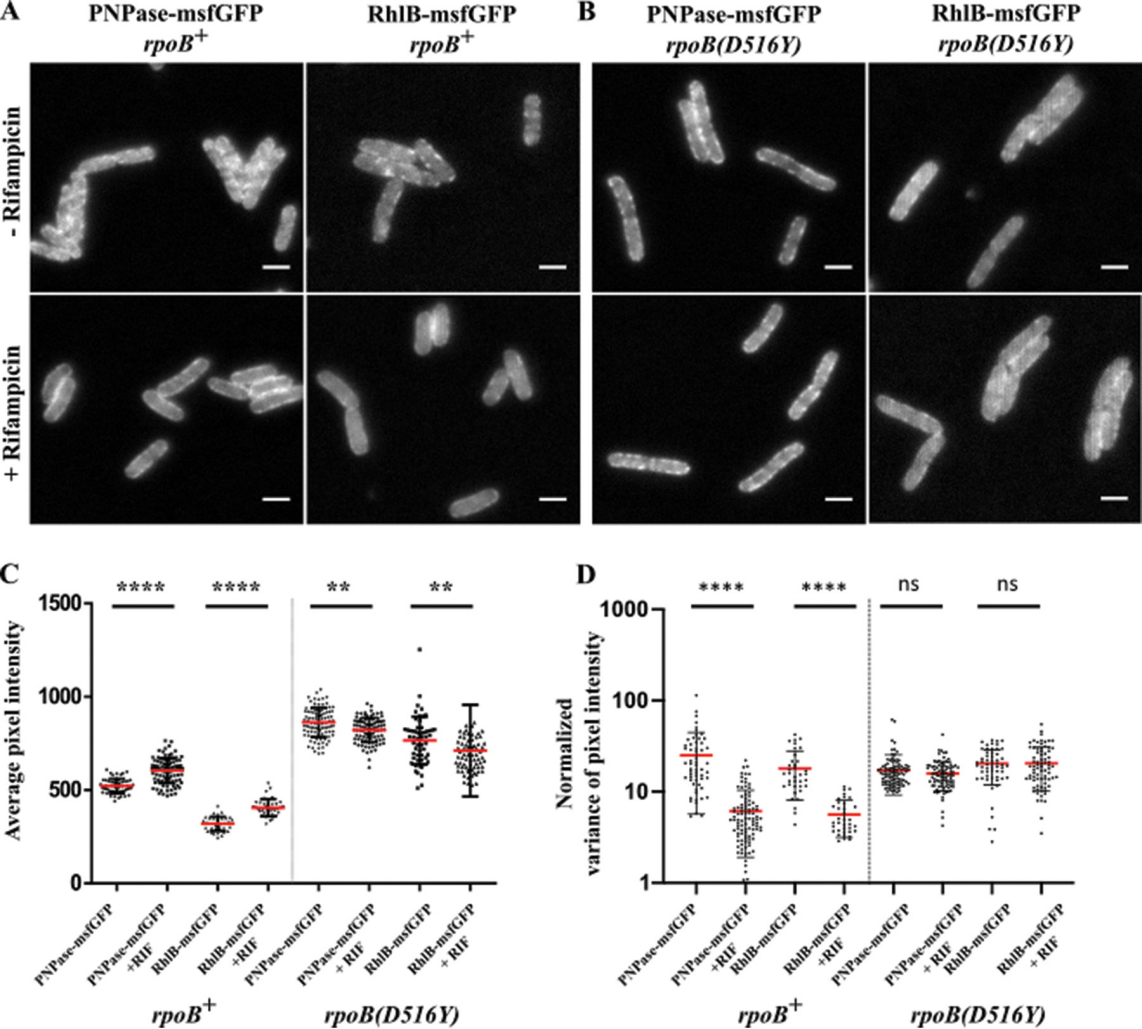
Inhibition of PNPase-msfGFP and RhlB-msfGFP punctum formation by rifampin. Strains SLM018 (PNPase-msfGFP, *rpoB^+^*), SLM024 (RhlB-msfGFP, *rpoB^+^*), SLM027 [PNPase-msfGFP, *rpoB*(*D516Y*)], and SLM029 [RhlB-msfGFP, *rpoB*(*D516Y*)] were grown to an OD_600_ of 0.5 to 0.6 in LB at 37°C. Cells were imaged before and after 30 min of treatment with 150 μg/ml of rifampin. (A and B) Epifluorescence images of PNPase-msfGFP and RhlB-msfGFP in the *rpoB^+^* background (A) and in the *rpoB*(*D516Y*) background (B). Bar, 2 μm. Average pixel intensity (C) and normalized variance in average pixel intensity (D) were calculated from line scans as described for Fig. 1. The red lines show means, and error bars show standard deviations. Data were collected from 40 to 100 line scans for each measurement. Statistical significance between conditions of treatment (with and without rifampin) was calculated using the nonparametric Mann-Whitney statistical test (****, *P < *0.0001; **, 0.001 < *P* < 0.01; ns, not significant [*P* > 0.05]).

To examine the localization and distribution of PNPase and RhlB, we constructed two strains, SLM018 and SLM024, expressing PNPase-msfGFP and RhlB-msfGFP, respectively. msfGFP is a monomeric derivative of superfolder GFP that minimizes aggregation and mislocalization (47). Both strains have growth rates comparable to that of their isogenic wild-type parent. We observed puncta of PNPase-msfGFP and RhlB-msfGFP at the periphery of the cell (Fig. 2A, top). After rifampin treatment, we detected a smooth distribution pattern (Fig. 2A, bottom). We also observed an increase in average pixel intensity (Fig. 2C). The smaller increase in msfGFP pixel intensity compared to mCherry is due to the 3-fold-higher rate of msfGFP maturation (48). There is a decrease in the normalized variance of PNPase-msfGFP and RhlB-msfGFP average pixel intensity after rifampin treatment (Fig. 2D). These results show that the membrane localization and distribution of PNPase-msfGFP and RhlB-msfGFP and the effect of rifampin treatment are the same as with RNase E-mCherry.

We next analyzed SLM027 and SLM029, which are rifampin-resistant *rpoB*(*D516*) strains encoding PNPase-msfGFP and RhlB-msfGFP, respectively (Table 1). In contrast to the *rpoB*^+^ strains, rifampin treatment did not result in dispersion of PNPase-msfGFP or RhlB-msfGFP (Fig. 2B). There was a small decrease in average pixel intensity and no difference in the normalized variance in average pixel intensity (Fig. 2C and D). These results show that rifampin treatment does not affect the clustering of RhlB-msfGFP and PNPase-msfGFP to form puncta in the *rpoB*(*D516Y*) background.

We constructed a fusion protein in which the RNase E sequence from position 568 to 592, corresponding to the MTS, was fused to the N terminus of msfGFP. Figure S1 shows that msfGFP localizes to the inner membrane, as evidenced by the ghost-like epifluorescence image and the localization to the surface of the cell in the TIRF image. As expected, treatment with rifampin resulted in an increase in average pixel intensity. There was, however, no significant change in the normalized variance in pixel intensity. This result shows that the MTS can target msfGFP to the inner membrane. The smooth, uniform distribution shows that the MTS by itself does not promote punctum formation and that membrane attachment does not aggregate msfGFP.

10.1128/mBio.01932-21.1 FIG S1 Characterization of the MTS-msfGFP fusion protein. MTS-msfGFP is a protein in which the RNase E sequence from position 568 to 592, corresponding to the MTS, was fused to the N terminus of msfGFP. MTS-msfGFP is expressed from the pLP120 plasmid, which was transformed into NCM3416 (SLP122 strain). pLP120 is a low-copy-number plasmid conferring spectinomycin resistance. Expression is under the control of a *lac* promoter. Cells were grown in LB with 40 μg/ml spectinomycin and 100 μM IPTG (isopropyl-β-d-thiogalactopyranoside) to mid-logarithmic phase; 1 to 2 μl of each culture was mounted on glass microscope slides with 1.2% (wt/vol) agarose pads. (A) Epifluorescence and TIRF images of the MTS-msfGFP fusion protein. The icon on the bottom right corner of each image shows the plane of focus. (B) Average pixel intensity and normalized variance in pixel intensity before and 30 min after the addition of rifampicin (150 μg/ml) were measured by quantification of line scans as for Fig. 1 and 2. Data were collected from 72 and 111 line scans for measurements with and without rifampin, respectively. Red horizontal lines show medians. Statistical significance was calculated using the nonparametric Mann-Whitney test (****, *P < *0.0001; ns, not significant). Download FIG S1, TIF file, 1.2 MB. Copyright © 2021 Hamouche et al. 2021 Hamouche et al. https://creativecommons.org/licenses/by/4.0/ This content is distributed under the terms of the Creative Commons Attribution 4.0 International license.

### Rifampin inhibition of clustering relaxes constraints on diffusion of the RNA degradosome.

Some membrane proteins have been shown to form puncta at the periphery of the cell. In B. subtilis, 65% of more than 200 proteins are localized in patchy patterns (49). Examples include SecA/SecY (50), MreB (51), Mdr (49), and RNase Y (45). The appearance and disappearance of RNase E-YFP puncta in E. coli over a period of seconds give the impression of movement (36). Photobleaching by TIRFm is a powerful method to measure relative rates of diffusion of membrane-associated proteins. Since only the membrane closest to the glass coverslip is excited in TIRFm, the cell is partitioned into illuminated and dark compartments. Rapid diffusion relative to the intrinsic rate of photobleaching results in slow photobleaching, since individual molecules spend only a short time in the illuminated field. In contrast, slow diffusion results in fast photobleaching. TIRFm photobleaching of membrane proteins results in biphasic curves that can be fitted using an initial high rate (*K_f_*) and a low rate (*K_s_*). *K_f_* and *K_s_* are not independent. However, *K_f_* can be thought of as the initial rate of photobleaching of molecules in the illuminated field, whereas *K_s_* is related to the rate of diffusion. Under carefully controlled conditions, *K_s_* can be used to measure relative rates of diffusion (36, 52). Previous work showed that rifampin treatment increased the rate of diffusion of RNase E-YFP (36).

**FIG 3:**
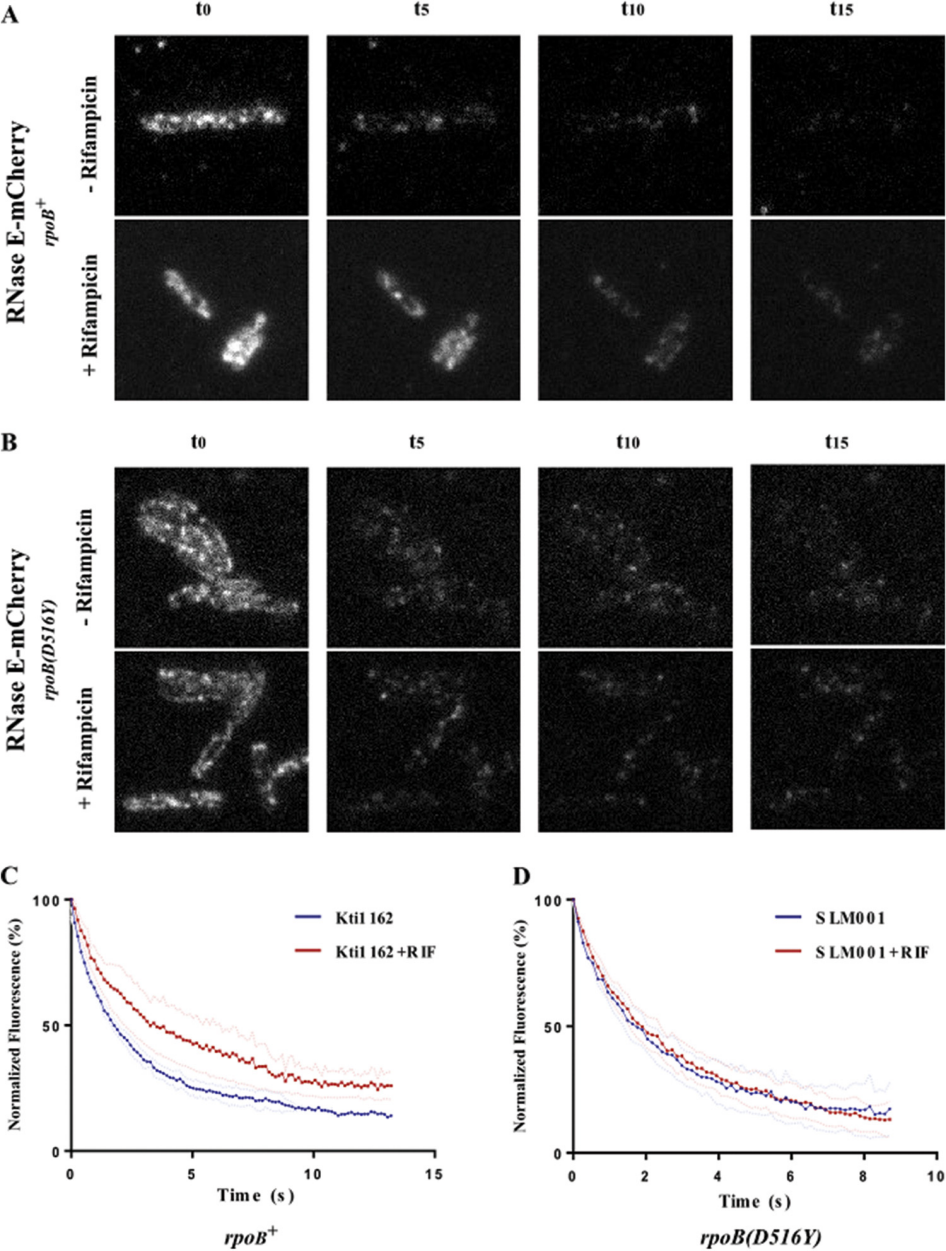
Photobleaching of RNase E-mCherry in the *rpoB^+^* and *rpoB*(*D516Y*) backgrounds. Cultures of Kti162 and SLM001 expressing RNase E-mCherry in the *rpoB^+^* and *rpoB*(*D516Y*) backgrounds, respectively, were grown to an OD_600_ of 0.5 to 0.6 in LB at 37°C. Images taken from 15-s TIRFm time-lapse videos filmed with no delay and a 100-ms exposure time before and after treatment with 150 μg/ml rifampin for 30 min. (A and B) Photobleaching of Kti162 (A) and SLM001 (B). (C and D) Quantification of TIRFm continuous photobleaching of Kti162 (C) and SLM001 (D). The graphs show the averaged percent normalized fluorescence intensities (blue and red dots), after background subtraction, of two independent fields of cells, before and after treatment with rifampin. Curve fits were performed using a biexponential decay model, where the mCherry rapidly bleaches with an initial intensity of 100 at time zero. Faint blue and red dotted lines show standard deviations.

We measured photobleaching of RNase E-mCherry in Kti162 and SLM001 before and after treatment with rifampin. Snapshots (Fig. 3A and B) suggest that photobleaching in the *rpoB^+^* strain is slower after treatment with rifampin. This was confirmed by quantification of the rate of photobleaching of a population of cells. The graphs in Fig. 3C and D show quantification of time-lapse videos in which photobleaching is continuous. Whereas rifampin treatment results in slower photobleaching in the *rpoB^+^* strain, there is no effect in the *rpoB*(*D516Y*) strain. We observed a similar effect of rifampin on RNase E-GFP photobleaching (Fig. S2). Table 2 shows the slow diffusion-limited rate of photobleaching (*K_s_*), the goodness of fit (*R*^2^), the standard error of the mean (SEM), and the number of measurements (*n*). These results show that the diffusion rate of RNase E-mCherry and RNase E-GFP expressed from the native *rne* locus under the control of endogenous regulatory elements increases with rifampin treatment. The *rpoB*(*D516Y*) mutation is validated as a control, since photobleaching is not affected by rifampin.

**TABLE 2:** Photobleaching measurements^*a*^

Strain	Fluorescent protein	*rpoB*	Without rifampin			With rifampin			*n*
			*K_s_* (1/s)	*R* ^2^	SEM	*K_s_* (1/s)	*R* ^2^	SEM	
Kti162	RNase E-mCherry	+	0.4356	0.9763	0.01106	0.2760	0.8223	0.02536	3
Kti164	RNase E-GFP	+	0.2493	0.9721	0.00655	0.1655	0.9302	0.00887	3
SLM001	RNase E-mCherry	*	0.4753	0.9955	0.01021	0.4375	0.9949	0.01059	3
SLM018	PNPase-msfGFP	+	0.03337	0.9987	0.00023	0.01510	0.9997	0.00027	3
SLM027	PNPase-msfGFP	*	0.03356	0.9998	0.00048	0.03660	0.9998	0.00040	2
SLM024	RhlB-msfGFP	+	0.3352	0.9993	0.01456	0.1024	0.9847	0.09171	3
SLM029	RhlB-msfGFP	*	0.2286	0.9579	0.01946	0.2109	0.9519	0.00812	2

a *K_s_* is the diffusion-limited slow rate constant, *R*^2^ is the goodness of fit, and SEM is the standard error of the curve fit. *n*, number of measurements. *, *rpoB*(*D516Y*) allele.

10.1128/mBio.01932-21.2 FIG S2 The diffusion rate of RNase E-GFP increases after rifampin treatment. Strain Kti164, which expresses RNase E-GFP, was grown to mid-log phase in LB at 37°C. (A) Images showing the photobleaching of RNase E-GFP were extracted from a 20-s TIRFm time lapse video (no delay; exposure time = 100 ms) with and without 30 min of rifampin treatment (150 μg/ml). (B) Averaged, background-subtracted, and normalized fluorescence values plotted versus time. Dotted lines show standard errors of curve fitting (SEM). Values from two-phase curve fitting are listed in Table 2. Download FIG S2, TIF file, 1.1 MB. Copyright © 2021 Hamouche et al. 2021 Hamouche et al. https://creativecommons.org/licenses/by/4.0/ This content is distributed under the terms of the Creative Commons Attribution 4.0 International license.

**FIG 4:**
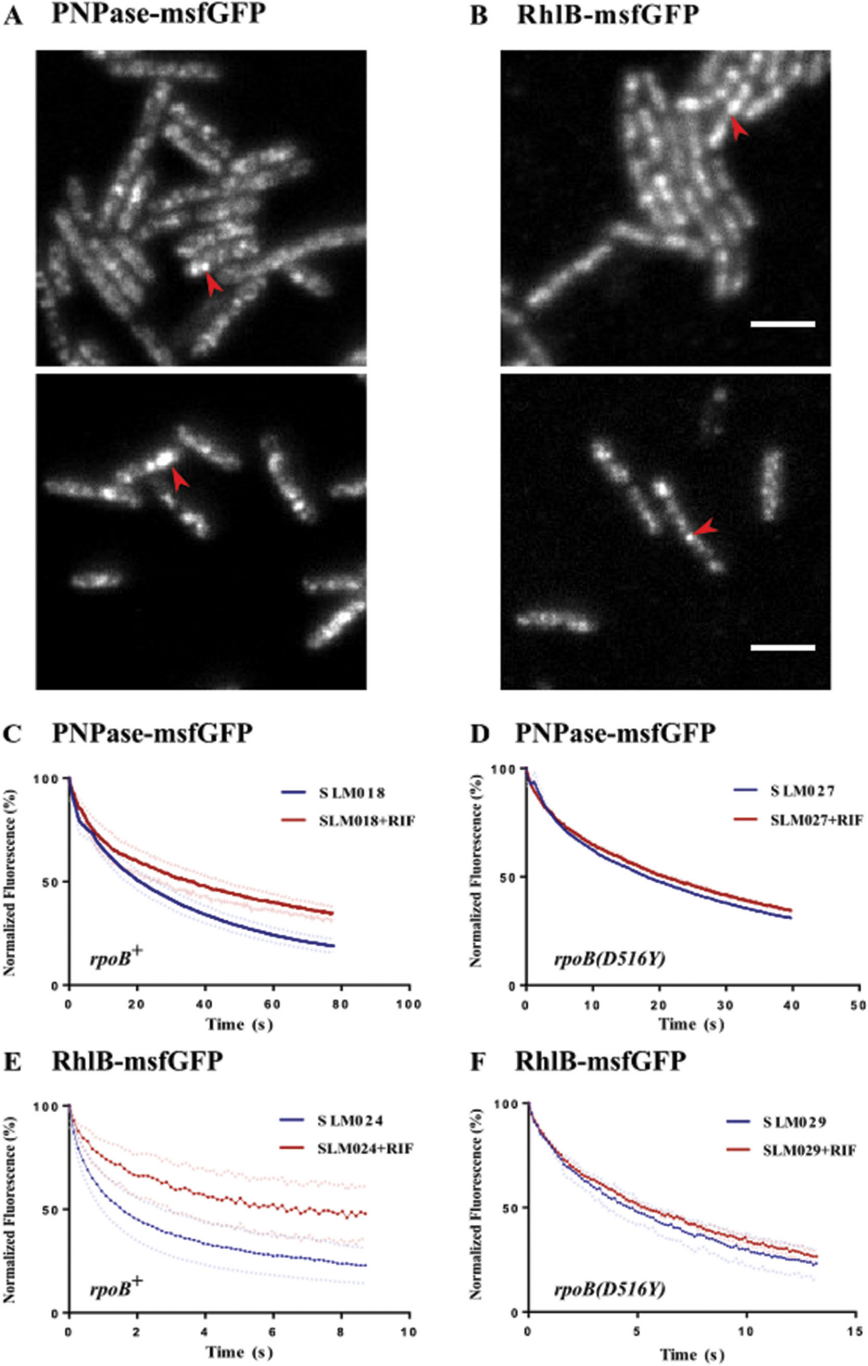
Photobleaching of PNPase-msfGFP and RhlB-msfGFP in the *rpoB^+^* and *rpoB*(*D516Y*) backgrounds. (A and B) PNPase-msfGFP (A) and RhlB-msfGFP (B). E. coli cells were grown in LB medium at 37°C and imaged by TIRFm. Bar, 5 μm. Red arrows indicate puncta. (C to F) Quantitative analysis of continuous photobleaching as described for Fig. 3. Photobleaching was performed before or 30 min after rifampin treatment (150 μg/ml). (C) SLM018 is PNPase-msfGFP and *rpoB^+^*. (D) SLM027 is PNPase-msfGFP and *rpoB*(*D516Y*). (E) SLM024 is RhlB-msfGFP and *rpoB^+^*. (F) SLM029 is RhlB-msfGFP and *rpoB*(*D516Y*).

In TIRFm imaging, PNPase-msfGFP and RhlB-msfGFP cluster in puncta (Fig. 4A and B), which are similar to RNase E-mCherry (Fig. 3). We used TIRFm photobleaching to measure the effect of rifampin treatment on diffusion. The rates of photobleaching of PNPase-msfGFP and RhlB-msfGFP decreased after rifampin treatment (Fig. 4C and E). In the *rpoB*(*D516Y*) background, there was no change (Fig. 4D and F). Table 2 shows the curve fitting constants obtained from these measurements. The diffusion rates of PNPase-msfGFP and RhlB-msfGFP increase after rifampin treatment, as is the case for RNase E-mCherry and RNase E-GFP. We also measured photobleaching of the MTS-msfGFP fusion protein before and after rifampin treatment (Fig. S3). As there is no effect on MTS-msfGFP, this result shows that the decrease in the rates of photobleaching of PNPase-msfGFP and RhlB-msfGFP after rifampin treatment is specific to components of the RNA degradosome.

10.1128/mBio.01932-21.3 FIG S3 MTS-msfGFP photobleaching. Photobleaching before and after treatment with rifampin (150 μg/ml). Cells were grown and mounted on agarose pads as for Fig. S1. TIRFm time-lapse videos were taken with no delay and 100 ms exposure. The graph shows quantitative analysis of continuous photobleaching as described for Fig. 3. The table gives values from two-phase curve exponential decay fitting. Download FIG S3, TIF file, 0.4 MB. Copyright © 2021 Hamouche et al. 2021 Hamouche et al. https://creativecommons.org/licenses/by/4.0/ This content is distributed under the terms of the Creative Commons Attribution 4.0 International license.

### Colocalization of RNA degradosome components.

**FIG 5:**
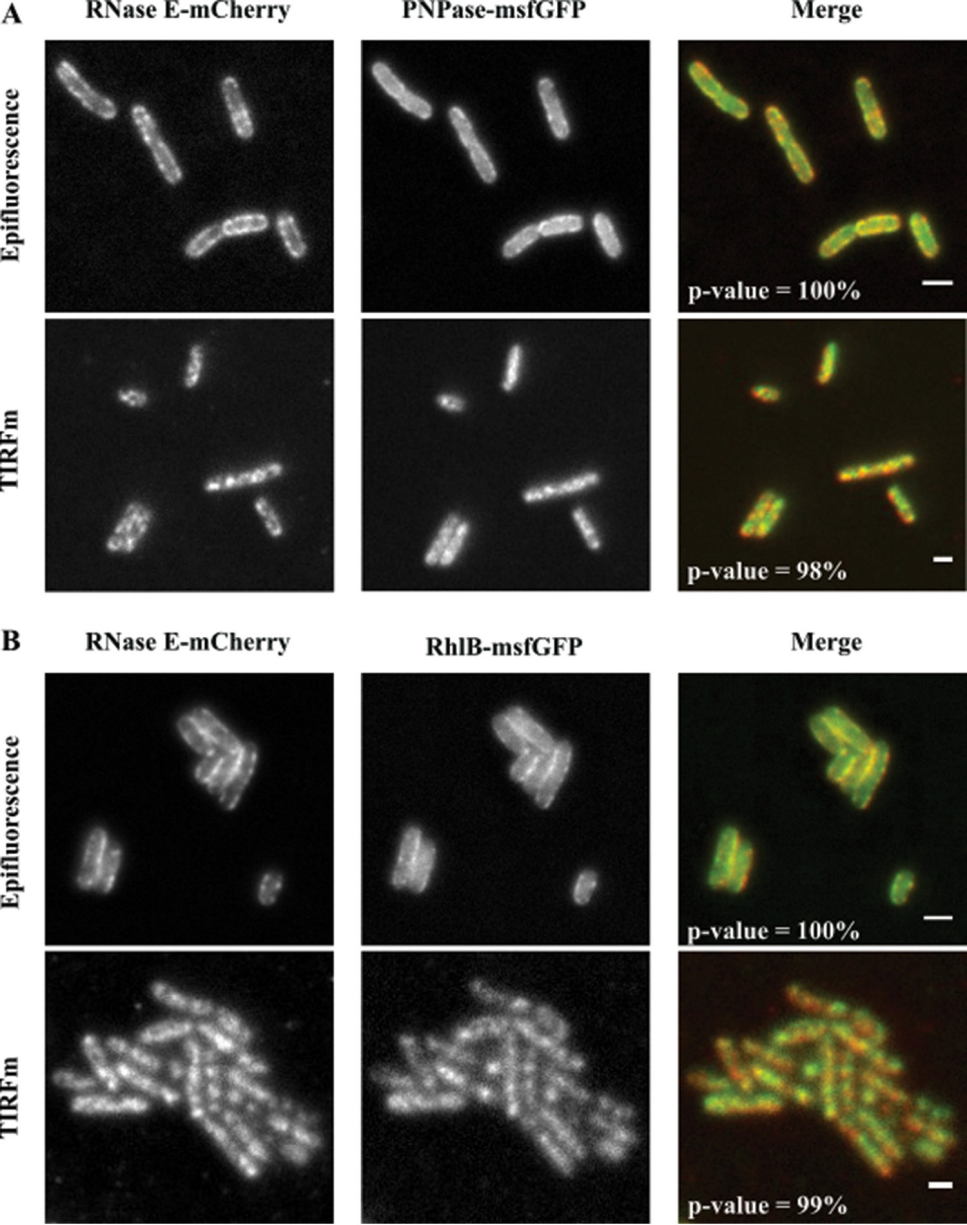
RNase E colocalizes with PNPase and RhlB. (A) E. coli strain SLM019 expressing RNase E-mCherry (left) and PNPase-msfGFP (middle) and merged images (right). (B) E. coli strain SLM025 expressing RNase E-mCherry (left) and RhlB-msfGFP (middle) and merged images (right). Cells were fixed with 1% formaldehyde and then imaged in epifluorescence (top) and TIRFm (bottom) illumination modes. After image background subtraction and conversion into 8-bit type, an analysis was performed using the ImageJ JACoP plugin (54). Costes’ randomization function was used to generate 1,000 scrambled images. Pearson’s correlation coefficient was calculated for each scrambled image and then compared to the original nonrandomized image. *P* values, expressed as percentages, are shown in the merged images. *P* values that exceed 95% are deemed statistically significant. Bar, 2 μm.

Previous work showed that membrane localization of RhlB depends on a direct protein-protein interaction with RNase E (36), and this is also likely for PNPase (37). Here, we used epifluorescence and TIRF microscopy to examine the colocalization of PNPase and RhlB with RNase E. Since PNPase and RhlB form puncta, we coexpressed RNase E-mCherry with RhlB-msfGFP (SLM025) or PNPase-msfGFP (SLM019). To investigate if the protein pairs colocalized, we fixed cells with 1% formaldehyde and imaged by epifluorescence and TIRF illumination. A merge of artificially colored red/green fields suggests that PNPase-msfGFP and RhlB-msfGFP colocalize with RNase E-mCherry, as evidenced by the yellow color (Fig. 5A and B). Several factors, including alignment of the images, noise, and the limit of resolution of light microscopy, which is about one-quarter the width of an E. coli cell, affect colocalization measurements. We therefore applied a pixel-by-pixel analysis of the RNase E-mCherry/PNPase-msfGFP and RNase E-mCherry/RhlB-msfGFP pairs. Figure S4 shows a graphical representation of the correlation in pixel intensity between the mCherry and msfGFP pairs. *Rr* values, which are a measure of the degree of correlation, are shown in the insets of the graphs (Fig. S4). The maximum value for Pearson’s coefficient (*Rr *= 1.000) indicates perfect correlation (52). Considering previous studies using Pearson’s coefficient in fluorescent-image analyses, *Rr* values in the range of 0.88 to 0.95 are strong support for colocalization of these proteins (52, 53).

10.1128/mBio.01932-21.4 FIG S4 Fluorescence intensity correlation graphs. Scatter plots of mCherry and msfGFP pixel intensities of the cells in Fig. 5. The plots show a pixel-by-pixel intensity correlation between RNase E-mCherry and PNPase-msfGFP (A) or RhlB-msfGFP (B) in two illumination modes: Epifluorescence (left) and TIRF (right). Pearson’s correlation coefficients (*Rr*), as described for Fig. 5, are shown at the top left of plots. Download FIG S4, TIF file, 2.1 MB. Copyright © 2021 Hamouche et al. 2021 Hamouche et al. https://creativecommons.org/licenses/by/4.0/ This content is distributed under the terms of the Creative Commons Attribution 4.0 International license.

We also performed a statistical analysis in which the micrographs were treated with Costes’ randomization function (54) to generate 1,000 scrambled images. Pearson’s correlation coefficients were calculated for each scrambled image compared to the nonrandomized image of the partner protein. As expected, the same results were obtained when scrambled mCherry images were compared to the msfGFP image and the scrambled msfGFP images were compared to the mCherry image. *P* values, expressed as percentages, are shown in the merged images (Fig. 5). *P* values of 100% in the epifluorescence images and 98 and 99% in the TIRFm images offer strong statistical support for the colocalization of RNase E-mCherry, PNPase-msfGFP, and RhlB-msfGFP.

We also analyzed epifluorescence and TIRF images of an RNase E-mCherry/AtpB-msfGFP pair (Fig. S5). AtpB is a subunit of the F_1_ ATPase, which is partly localized to the inner cytoplasmic membrane. Membrane-associated AtpB is visible in epifluorescence (Fig. S5, top) and TIRF (bottom) imaging. Visual inspection of the micrographs in Fig. S5A suggests little overlap of RNase E-mCherry puncta and AtpB-msfGFP puncta. Correlation coefficients of less than 0.80 (Fig. S5B) are consistent with little or no overlap localization of RNase E-mCherry and AtpB-msfGFP.

10.1128/mBio.01932-21.5 FIG S5 AtpB localization. (A) E. coli strain SLM036 expressing RNase E-mCherry (left) and AtpB-msfGFP (middle), and merged images (right). Cells were imaged in epifluorescence (top) and TIRF (bottom) illumination modes. Bar, 2 μm. (B) Scatter plots and Pearson’s correlation coefficients (*Rr*) as described for Fig. 5 and Fig. S4. Download FIG S5, TIF file, 2.8 MB. Copyright © 2021 Hamouche et al. 2021 Hamouche et al. https://creativecommons.org/licenses/by/4.0/ This content is distributed under the terms of the Creative Commons Attribution 4.0 International license.

Our epifluorescence images (Fig. 2 and 5) suggest that in addition to membrane localization, a proportion of PNPase is cytoplasmic. Modeling based on known quaternary structures and protein-protein interactions shows that 1 trimer of PNPase can bind to 1 protomer of RNase E (3:1) (17). Measurements of fluorescent protein levels in chemically fixed cells gave a 5.5:1 ratio of PNPase to RNase E (55), thus suggesting that there are nearly equivalent levels of RNA degradosome-associated PNPase and free PNPase. This estimate is consistent with absolute protein synthesis rates measured by ribosome profiling, which shows an excess of PNPase synthesis relative to RNase E (4.4:1) (56). The existence of free PNPase is further supported by pulldown experiments in which some, but not all, PNPase copurifies with RNase E (e.g., see Fig. 2 in reference 39).

PNPase-msfGFP photobleaching is 5- to 10-fold slower than RhlB-msfGFP photobleaching (Table 2). We therefore measured *in vitro* photobleaching of purified RNA degradosomes containing PNPase-msfGFP or RhlB-msfGFP (Fig. S6 and S7). Since there is no difference in the intrinsic sensitivity of the purified complexes to photobleaching *in vitro*, the slow photobleaching of PNPase-msfGFP must be due to the structure and/or dynamics of the RNA degradosome *in vivo*. Possible explanations include (i) geometry of the RNA degradosome on the inner cytoplasmic membrane, in which PNPase-msfGFP is further from the glass slide than RhlB-msfGFP, resulting in lower excitation levels, and (ii) an exchange between degradosome-associated PNPase-msfGFP and cytoplasmic PNPase-msfGFP, which would serve as an additional pool of photobleachable molecules. This phenomenon was not investigated further.

10.1128/mBio.01932-21.6 FIG S6 Nanoscale photobleaching of purified RNA degradosome. (A) Silver-stained gel of native RNA degradosome purified by affinity of RNase E containing a C-terminal FLAG tag (strains SAJ259 and SAJ260 corresponding to PNPase-msfGFP and RhlB-msfGFP, respectively). Size markers (M1) are indicated to the left. E1 and E2 are fractions eluted with FLAG peptide. A protein standard (M2) was used to estimate concentration of RNase E. The asterisks indicate LDH subunits that bind to the anti-FLAG beads due to autogenous FLAG epitopes. RhlB-msfGFP copurifies with a minor contaminant. (B) Photobleaching of 1-μl droplets of affinity-purified RNA degradosome (∼2 ng RNase E) was measured by time lapse videos using wide-field illumination. The Cy5-oligoRNA was used to center the droplet. Photobleaching is quantified by integrating the intensity of the entire field in the time-lapse video. The green-to-black transition in the time-lapse video is an illustration. It does not represent real data. Download FIG S6, TIF file, 1.3 MB. Copyright © 2021 Hamouche et al. 2021 Hamouche et al. https://creativecommons.org/licenses/by/4.0/ This content is distributed under the terms of the Creative Commons Attribution 4.0 International license.

10.1128/mBio.01932-21.7 FIG S7 *In vitro* rates of photobleaching of purified RNA degradosome. Representative photobleaching curves of RNA degradosomes labelled with PNPase-msfGFP (A) or RhlB-msfGFP (B) purified as described for Fig. S6A. The curves are derived from a time-lapse video as described for Fig. S6B. (C) The table shows fast (*K_f_*) and slow (*K_s_*) rate constants from two-phase curve fitting, standard deviation (sd), and number of measurements (n). Download FIG S7, TIF file, 1.1 MB. Copyright © 2021 Hamouche et al. 2021 Hamouche et al. https://creativecommons.org/licenses/by/4.0/ This content is distributed under the terms of the Creative Commons Attribution 4.0 International license.

### Kasugamycin treatment inhibits RNA degradosome clustering.

**FIG 6:**
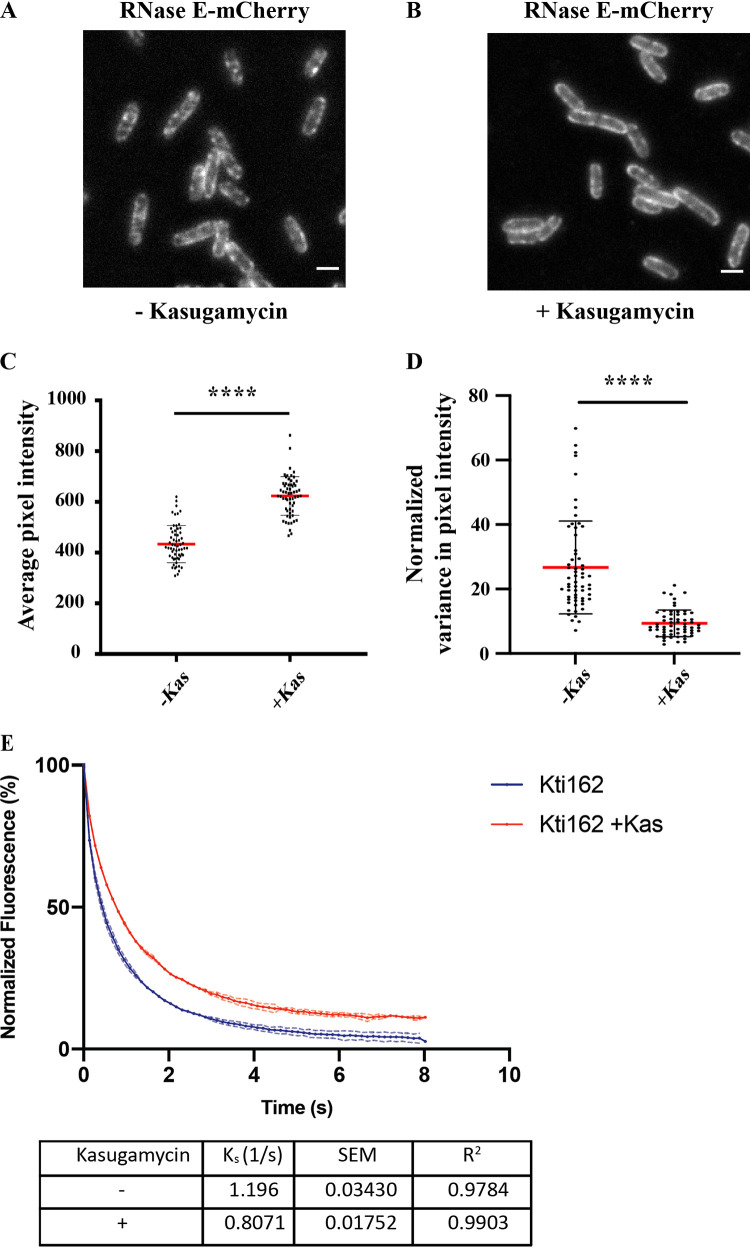
Kasugamycin (Kas) treatment inhibits formation of RNase E puncta and increases diffusion rate. Kti162 strain expressing RNase E-mCherry was grown to an OD_600_ of 0.5 to 0.6 in LB at 37°C. (A and B) Epifluorescence micrographs of RNase E-mCherry showing the distribution of RNase E before (A) and after (B) treatment with kasugamycin for 30 min. Bar, 2 μm. Average pixel intensity (C) and variance in average pixel intensity (D) were determined as described for Fig. 1. Sixty line scans of cells before and 66 line scans of cells after treatment with kasugamycin were analyzed to generate graphs of average and normalized variance in pixel intensity. Red horizontal lines show means, and the error bars represent standard deviations. Statistical significance was calculated using the nonparametric Mann-Whitney test (****, *P < *0.0001). (E) The graph shows the averaged, background subtracted, and normalized fluorescence values plotted versus time (seconds). Two fields of cells were analyzed and curves were fitted as described in Fig. 3. Dashed lines show standard errors (SEM). The table shows the values calculated from the curve fitting.

We asked if the formation of RNA degradosome puncta involves a direct interaction with mRNA by treating cells with kasugamycin, which results in inhibition of translation of canonical mRNAs with a 5′ leader containing a Shine-Dalgarno sequence, arrest of growth, and low-level translation of leaderless mRNA (57). Continued transcription results in the degradation of ribosome-free mRNA and the maturation of tRNA and rRNA precursors (43). Figure 6 shows that kasugamycin inhibits formation of RNA degradosome puncta. As expected, there was an increase in average pixel intensity due to maturation of mCherry (Fig. 6C). Statistical support for decreased normalized variance in pixel intensity is quantitative evidence for the inhibition of RNA degradosome clustering in a population of cells (Fig. 6D). Figure 6E shows that the rate of photobleaching of RNase E-mCherry decreased after kasugamycin treatment, which is consistent with an increase in the diffusion rate of the RNA degradosome. These results show that the degradation of ribosome-free mRNA and the maturation of tRNA and rRNA precursors in the presence of kasugamycin is not associated with the formation of RNA degradosome puncta.

We also measured the effect of kasugamycin on the photobleaching of AtpB-msfGFP. Figure S8 shows that kasugamycin treatment has no effect on the rate of photobleaching of AtpB-msfGFP. We therefore conclude that the effect of kasugamycin is specific to the dynamics of the RNA degradosome. The effects of kasugamycin treatment on RNA degradosome clustering and diffusion are very similar to the effects of rifampin treatment. Kasugamycin treatment has been shown to result in the absence of detectable polyribosomes (58). Since mRNA synthesis continues in the presence of kasugamycin (57), these results strongly suggest that the clustering of RNA degradosomes to form puncta depends on the presence of polyribosomes.

10.1128/mBio.01932-21.8 FIG S8 AtpB-msfGFP photobleaching. The graph shows photobleaching in the absence or presence of kasugamycin (Kas). The table shows fast (*K_f_*) and slow (K_s_) rate constants from two phase curve fitting, standard error (SEM), and fit (*R*^2^). Download FIG S8, TIF file, 0.4 MB. Copyright © 2021 Hamouche et al. 2021 Hamouche et al. https://creativecommons.org/licenses/by/4.0/ This content is distributed under the terms of the Creative Commons Attribution 4.0 International license.

### Chloramphenicol treatment results in large clusters of RNA degradosomes (foci).

**FIG 7:**
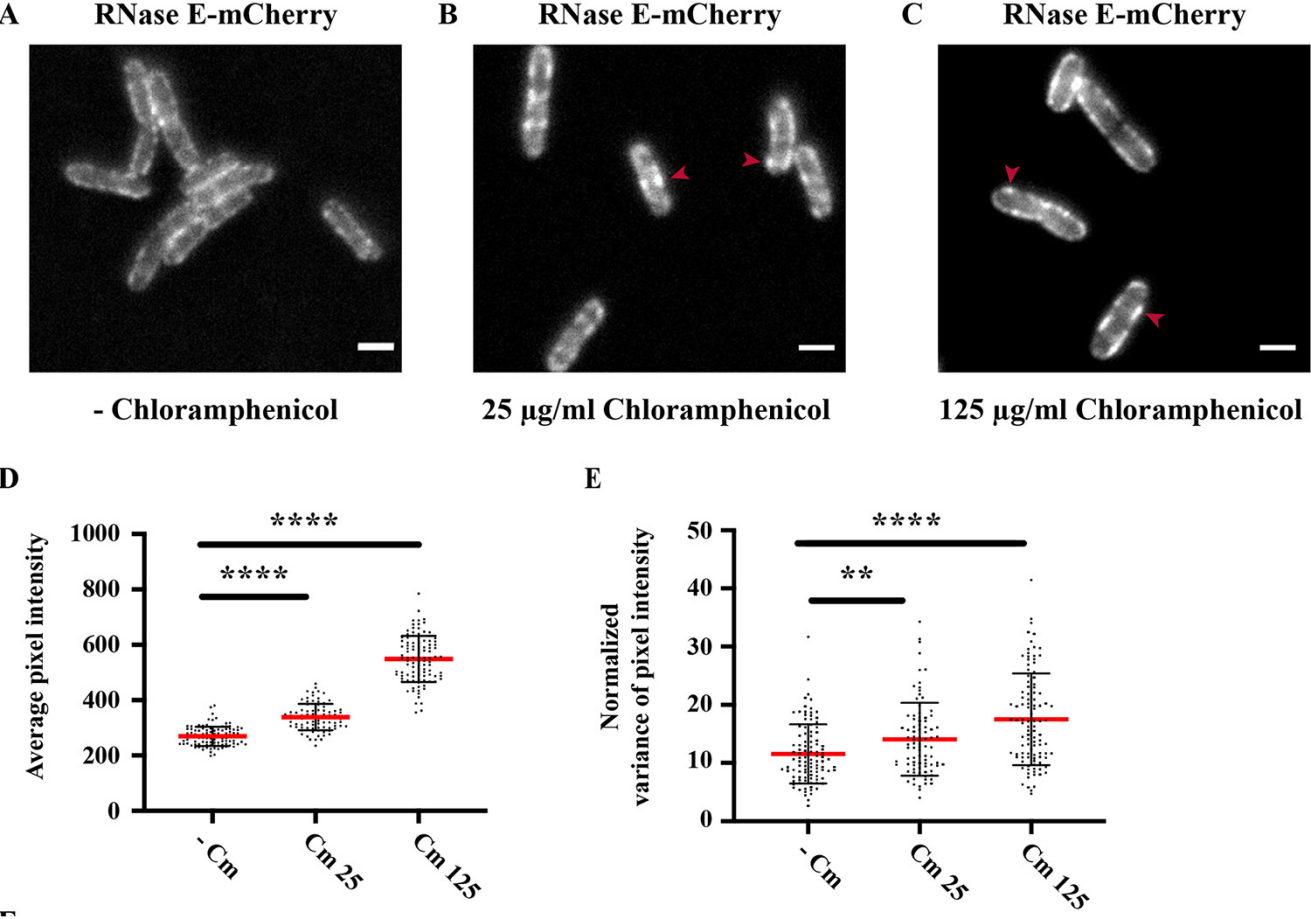
Chloramphenicol treatment results in focus formation. Kti162 expressing RNase E-mCherry was grown to an OD_600_ of 0.5 to 0.6 in LB at 37°C. (A to C) Epifluorescence micrographs of RNase E-mCherry. The images show the distribution of RNase E before (A) and after (B and C) treatment with 25 and 125 μg/ml chloramphenicol for 30 min. Bar, 2 μm. Average pixel intensity (D) and normalized variance in average pixel intensity (E) were determined as described in Fig. 1. Red horizontal lines mark the means. Data were collected from 88 to 104 line scans for each measurement. Statistical significance was calculated using the nonparametric Mann-Whitney test (****, *P < *0.0001; **, 0.001 < *P* < 0.01).

We tested the effect of another protein synthesis inhibitor on the formation of RNA degradosome puncta on the inner cytoplasmic membrane. Chloramphenicol inhibits peptide bond synthesis, thus freezing translation elongation (59). Figure 7A to C shows epifluorescent images of cells expressing RNase E-mCherry in the absence or presence of 25 or 125 μg/ml chloramphenicol. Although cell growth was inhibited on agar plates at the lower concentration, the higher concentration is often used with liquid media. The images reveal large clusters of RNA degradosomes that are distinct from the membrane-associated puncta seen under normal growth conditions (Fig. 7B and C, red arrows). Average pixel intensity and normalized variance in pixel intensity (Fig. 7D and E) are consistent with the formation of large clusters, which we refer to as foci. The increase in average pixel intensity is partly due to the maturation of mCherry after the inhibition of protein synthesis. However, the higher average pixel intensity at 125 μg/ml chloramphenicol than at 25 μg/ml suggests an additional effect, which could be due to the formation of foci. The increase in the normalized variance of pixel intensity at both concentrations of chloramphenicol is consistent with the localization of a large proportion of RNA degradosomes into a few foci. Taken together, these results show that the inhibition of translation elongation results in the formation of RNA degradosome clusters that are larger and brighter than puncta seen in untreated cells.

We were not able to make photobleaching measurements of chloramphenicol-treated cells, because there was no detectable RNase E-mCherry signal in TIRF microscopy. Since TIRF illuminates only molecules on the surface of the cell closest to the glass coverslip (60), this result suggests that RNase E-mCherry is no longer attached to the inner cytoplasmic membrane. As a control, we imaged RNase E-mCherry after chloramphenicol treatment in HILO (highly inclined and laminated optical) mode (Fig. S9), which is a variant of TIRF that permits imaging the interior of the cell. The signal in HILO mode shows that RNase E-mCherry is present in the cell after chloramphenicol treatment. These results strongly suggest that the RNA degradosome moves from the periphery of the cell to the interior after chloramphenicol treatment.

10.1128/mBio.01932-21.9 FIG S9 HILO images of RNase E-mCherry after treatment with chloramphenicol. Download FIG S9, TIF file, 1.3 MB. Copyright © 2021 Hamouche et al. 2021 Hamouche et al. https://creativecommons.org/licenses/by/4.0/ This content is distributed under the terms of the Creative Commons Attribution 4.0 International license.

## DISCUSSION

Epifluorescence and TIRF imaging of cells expressing the RNase E-mCherry and PNPase-msfGFP or RhlB-msfGFP shows that these proteins colocalize in puncta on the inner cytoplasmic membrane. Pearson’s correlation coefficients in a pixel-by-pixel comparison of epifluorescence and TIRF images range from 0.88 to 0.96. A statistical analysis employing Costes’ randomization function to generate scrambled images yielded *P* values of ≥98%. As a control, analysis of images of the RNase E-mCherry and AtpB-msfGFP, another inner membrane protein, resulted in Pearson’s correlation coefficients of less than 0.80. The statistical analysis together with the AtpB-msfGFP control is important experimental evidence for the association of RNase E, PNPase, and RhlB as components of the RNA degradosome in living cells.

Epifluorescence measurements show that rifampin treatment results in the dispersion of puncta containing RNase E, PNPase, and RhlB. Photobleaching measurements by TIRFm show that rifampin treatment relaxes constraints on the diffusion of RNase E, PNPase, and RhlB. Punctum formation is thus due to clustering of the RNA degradosomes. There are three arguments against the hypothesis that RNA degradosome puncta are an artifact of fluorescence protein aggregation. (i) Punctum formation has been seen with fusions of four different fluorescence proteins (YFP, GFP, mCherry, and msfGFP) to three different components of the RNA degradosome (RNase E, PNPase, and RhlB). (ii) With the exception of the MTS-msfGFP control, all fusion proteins in this work were expressed from single-copy chromosomal constructs at the *rne*, *pnp*, or *rhlB* locus under the control of endogenous expression signals. (iii) The dispersion of puncta by rifampin treatment argues that RNA degradosome clustering involves an additional factor whose presence depends on transcription.

**FIG 8:**
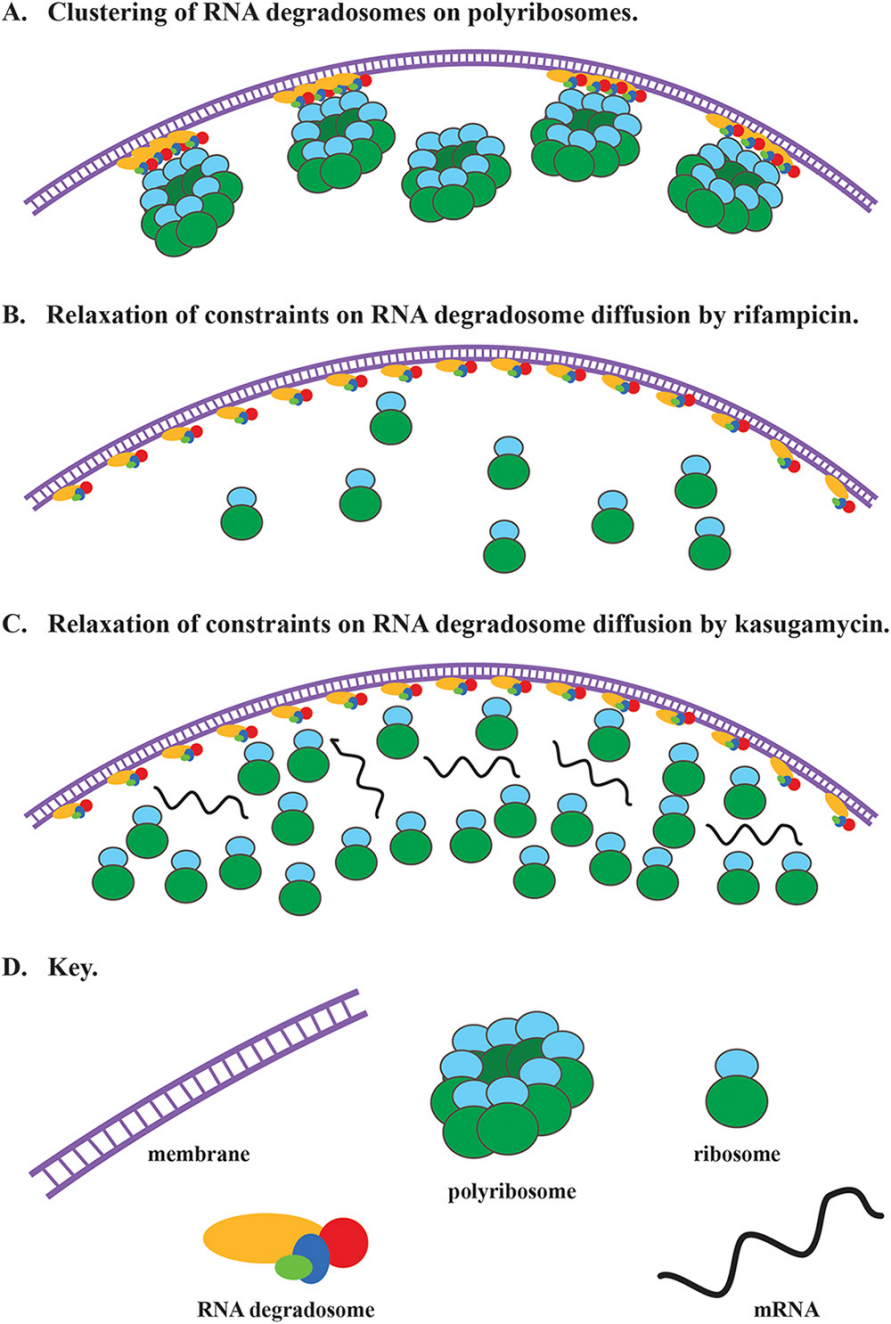
Model for clustering of RNA degradosome by polyribosomes. (A) Multiple RNA degradosomes associate transiently with polyribosomes to scan for exposed regions of mRNA that can be cleaved by RNase E to initiate degradation. (B) Disruption of polyribosomes after depletion of mRNA by rifampin treatment results in random distribution of RNA degradosomes. (C) Disruption of polyribosomes after inhibition of translation initiation by kasugamycin treatment results in random distribution of RNA degradosomes. (D) Key showing the inner cytoplasmic membrane, RNA degradosomes, polyribosomes, 70S ribosomes, and mRNA.

Kasugamycin treatment results in dispersion of RNA degradosome puncta and relaxation of constraints on diffusion. Although this effect is the same as that observed with rifampin, the mechanism is necessarily different. Figure 8A shows a cartoon in which we propose that RNA degradosomes cluster upon binding to a polyribosome to form puncta. Figure 8B and C show the outcome of rifampin and kasugamycin treatment, respectively. In Fig. 8B, polyribosomes and mRNA are absent due the degradation of mRNA, and there are fewer ribosomes due to the degradation of rRNA during rifampin treatment (43). In Fig. 8C, free ribosomes and mRNA are shown after kasugamycin treatment, since transcription continues and rRNA is not degraded (43, 57). The result of kasugamycin treatment shows that processing of tRNA and rRNA precursors, which continues (43), and degradation of ribosome-free mRNA do not result in the formation of puncta. Although ribosomes are freely diffusible, experimental work has shown that polyribosomes are constrained in a glass-like state (61, 62). Puncta could therefore be the result of the capture of slow-moving polyribosomes by RNA degradosomes. Our results strongly suggest that the puncta are sites of degradation of polyribosomal mRNA.

The inhibition of translation by chloramphenicol results in large clusters (foci) of RNase E-mCherry that are distinct from the puncta observed in untreated cells. The loss of an RNase E-mCherry signal in TIRF mode after chloramphenicol treatment strongly suggests that RNA degradosomes in the foci are not membrane attached. Recent work has shown that nitrogen starvation in E. coli triggers a stress response that results in the formation of foci containing RNA degradosomes and the RNA chaperone Hfq (63, 64). By analogy, we propose that the foci formed by chloramphenicol treatment are part of a stress response induced by the inhibition of translation elongation. We recently showed that rRNA is mostly stable after treatment with chloramphenicol, which contrasts with the massive degradation of rRNA after treatment with rifampin (43). The formation of foci could stockpile RNA degradosomes as part of a mechanism that protects inactive polyribosomes from degradation. Future work on characterization of the localization, composition, and dynamics of the chloramphenicol-induced RNA degradosome foci could give new insight into how the mRNA degradation machinery is regulated under stress conditions.

Biological systems are not engineered, they evolve, and conservation of RNase E homologues throughout the *Proteobacteria* emphasizes their importance in evolutionary fitness (4, 17). Protein sequence comparisons have shown that RNase E homologues in the gammaproteobacteria descended vertically from an ancient proto-RNase E that had a large noncatalytic region containing an MTS (18). Clustering of RNA degradosomes on polyribosomes could contribute to selectivity or efficiency in the initiation of mRNA degradation. Since bacteria are continually challenged by changes in the environment, the accuracy and speed with which gene expression is reprogrammed and fine-tuned are critical for survival of a population of cells. When bacterial cells are considered as a system, components such as the RNA degradosome interact with other components involved in the regulation of gene expression. Since mutations that affect RNA degradosome structure, function, or cellular localization could disrupt the correct functioning of other components, there is strong selective pressure to conserve its normal function(s). For this reason, we believe that RNA processing and mRNA degradation involving RNA degradosomes attached to the inner cytoplasmic membrane are conserved features of gammaproteobacterial cell biology.

## MATERIALS AND METHODS

### Media and strains.

Liquid medium (LB) and agar (LA) plates were prepared as described elsewhere (65). Strains used in this work are listed in Table 1. NCM3416 was the parent strain used for λ*red* recombination as described elsewhere (36, 66). Briefly, DNA templates encoding C-terminal mCherry and msfGFP fusions were generated by crossover PCR with an *frt-cat-frt* cassette. The resulting products were transformed into NCM3416/pKD46 and selected at 37°C on LB plates containing 12.5 μg/ml chloramphenicol. Recombinants were colony purified. The constructs were genetically purified by P1*_vir_* transduction into NCM3416. The *cat* cassette was removed by transformation with pCP20, which encodes the FLP recombinase (66). Constructs were validated by PCR amplification of genomic DNA, sequencing, and fluorescence microscopy. SLM019 and SLM025 were obtained by P1 transduction into Kti162. The *rpoB*(*D516Y*) mutation was selected as a spontaneous mutation of the NCM3416 strain, as described elsewhere (43).

### Microscopy and photobleaching.

TIRFm photobleaching experiments were carried out as described in reference 36. All epifluorescence and TIRFm acquisitions were taken independently and at least in duplicate for each strain and type of experiment. Images were analyzed using ImageJ v.1.38 (National Institutes of Health) (67, 68). Quantitative analyses of TIRFm photobleaching data to determine relative diffusion rates were performed as described in reference 52. The image analysis of colocalization experiments was carried out using ImageJ and the JACoP plugin (54). Graphic processing and two-tailed *t* tests for the statistical analysis were performed using GraphPad Prism v.8.0 with an alpha level of 5%.

### Affinity purification of native RNA degradosome.

The affinity purification protocol was adapted from procedures described in references 39 and 69. Buffers are described therein.

Additional experimental detail is given in Text S1.

10.1128/mBio.01932-21.10 TEXT S1 Cell imaging growth conditions, wide-field epifluorescence, TIRFm imaging, affinity purification of native RNA degradosome, and nanoscale photobleaching *in vitro*. Download Text S1, DOCX file, 0.01 MB. Copyright © 2021 Hamouche et al. 2021 Hamouche et al. https://creativecommons.org/licenses/by/4.0/ This content is distributed under the terms of the Creative Commons Attribution 4.0 International license.
