# Supplementary figures and images for: Polyribosome-Dependent Clustering of Membrane-Anchored RNA Degradosomes To Form Sites of mRNA Degradation in Escherichia coli
Source: mBio. 2021 Sep 7;12(5):e01932-21. doi: 10.1128/mBio.01932-21 (PMC8546579; doi:10.1128/mBio.01932-21)

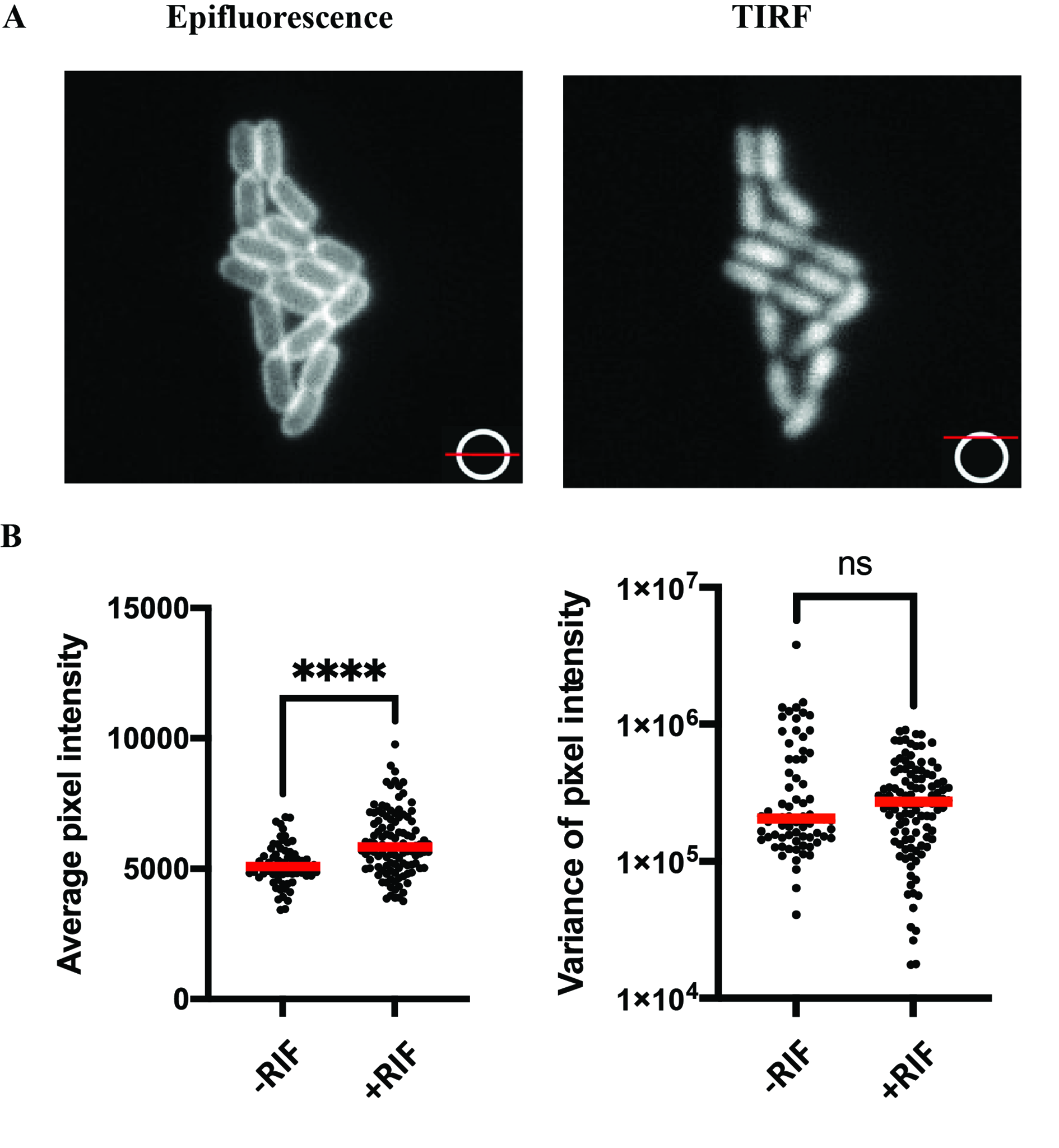

Supplement: FIG S1 [file mbio.01932-21-sf001.tif]

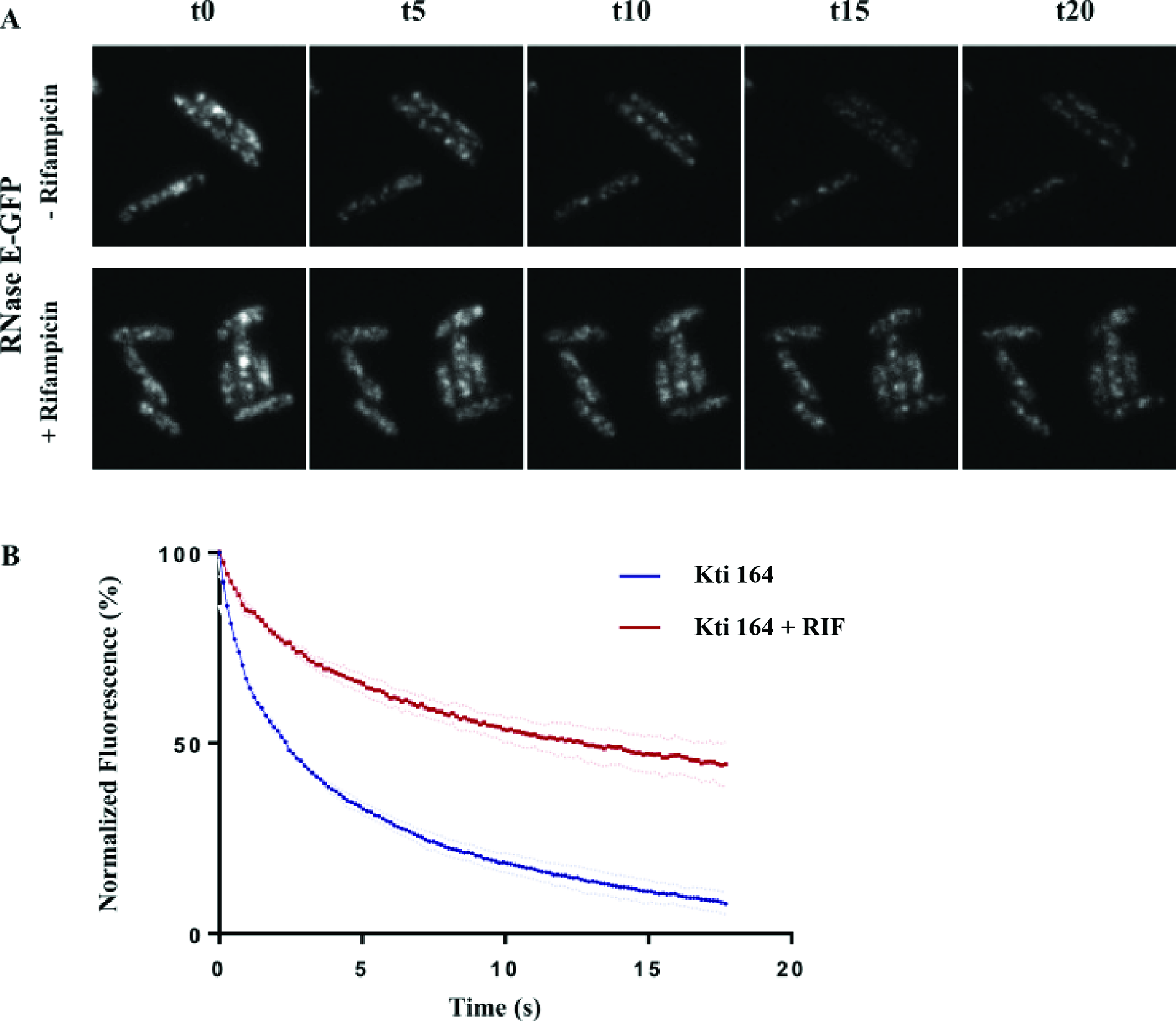

Supplement: FIG S2 [file mbio.01932-21-sf002.tif]

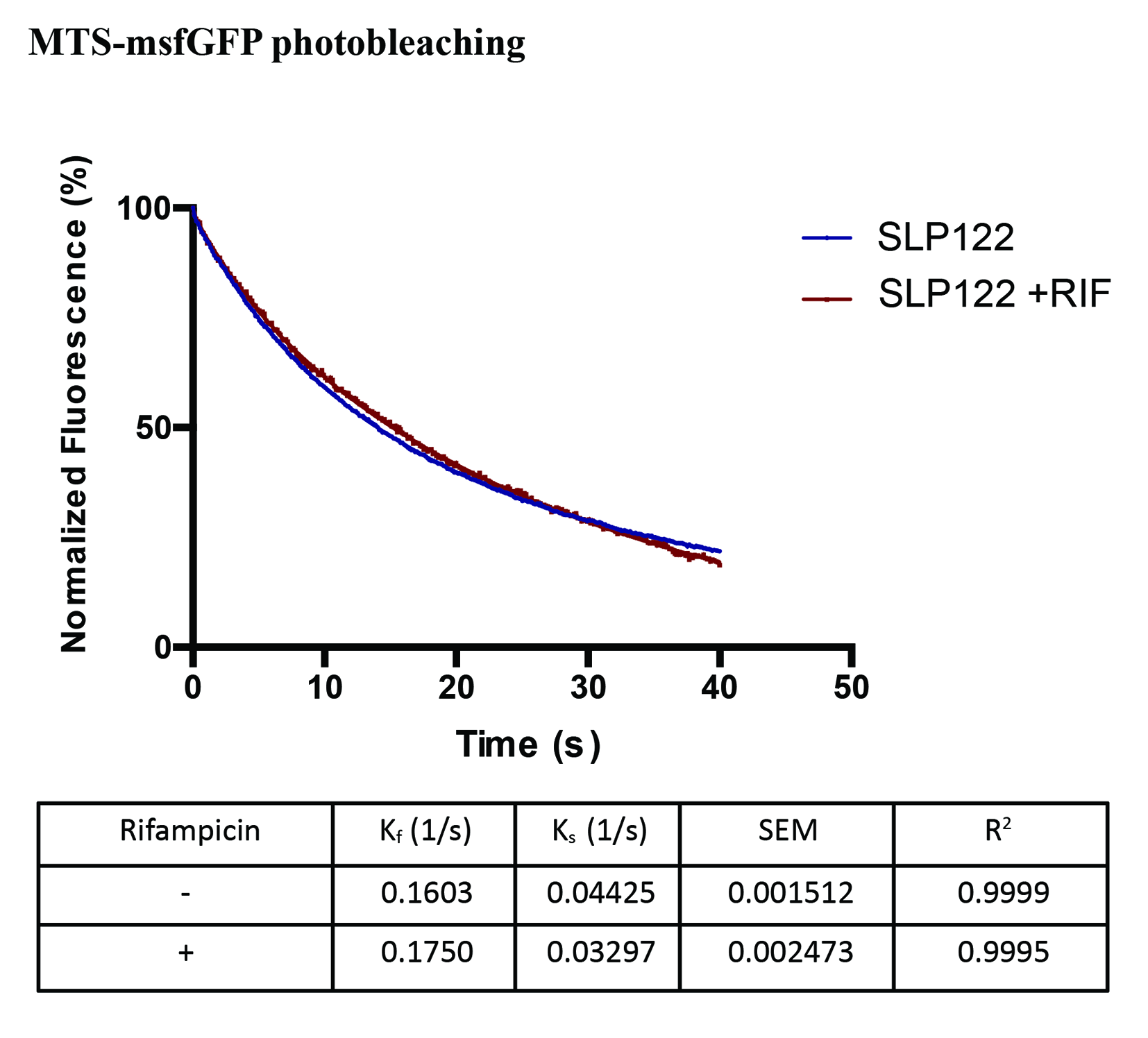

Supplement: FIG S3 [file mbio.01932-21-sf003.tif]

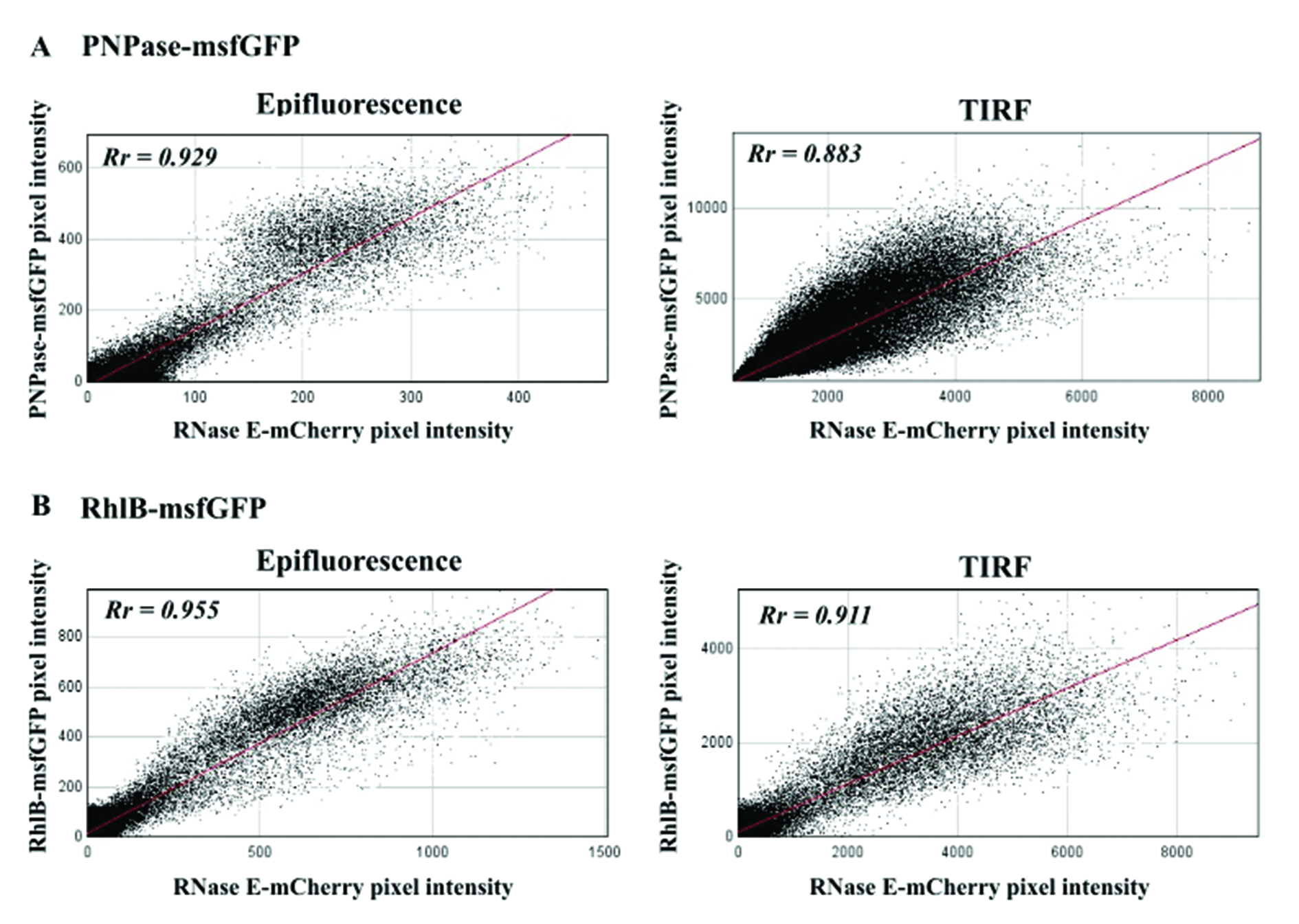

Supplement: FIG S4 [file mbio.01932-21-sf004.tif]

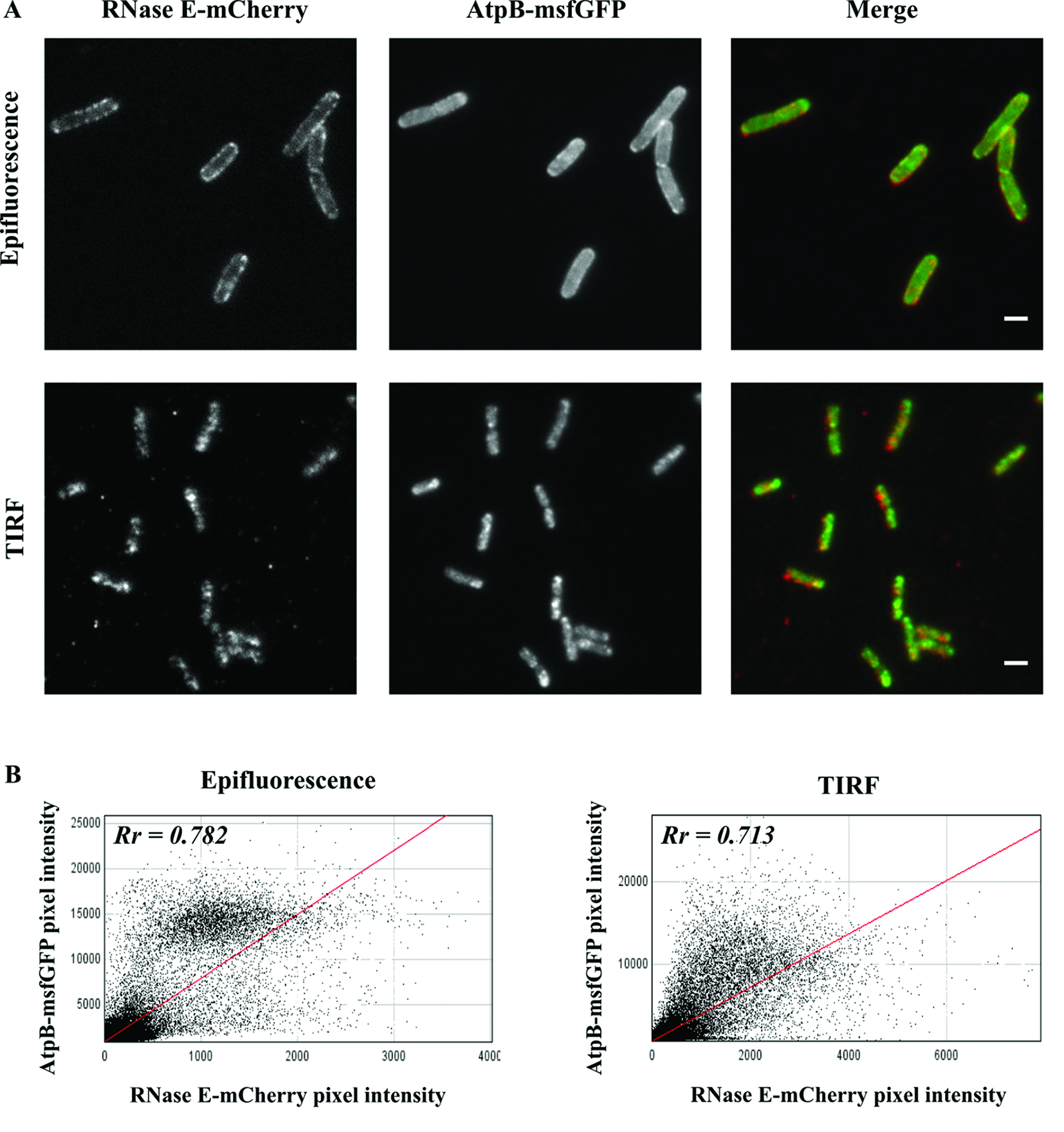

Supplement: FIG S5 [file mbio.01932-21-sf005.tif]

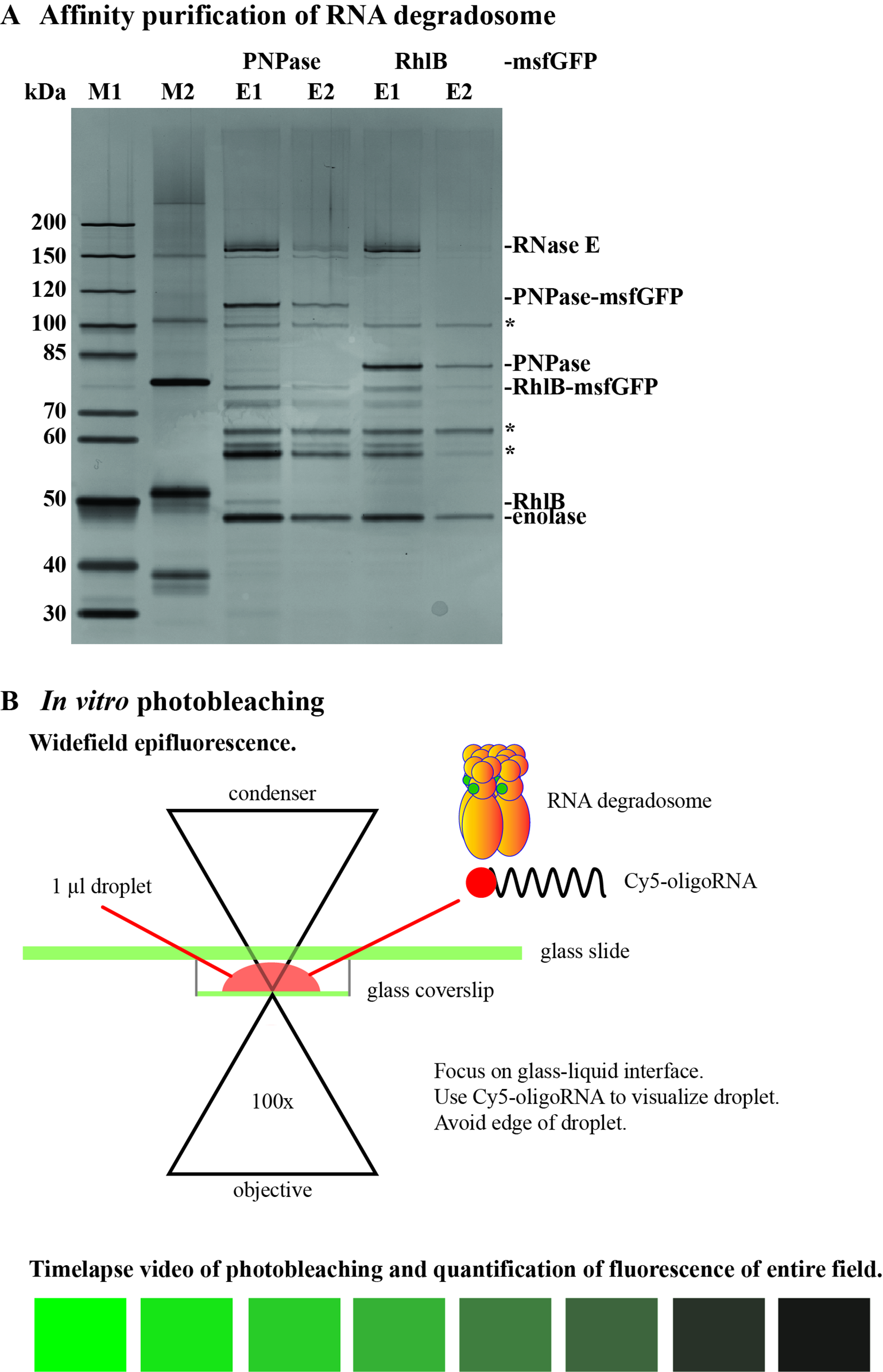

Supplement: FIG S6 [file mbio.01932-21-sf006.tif]

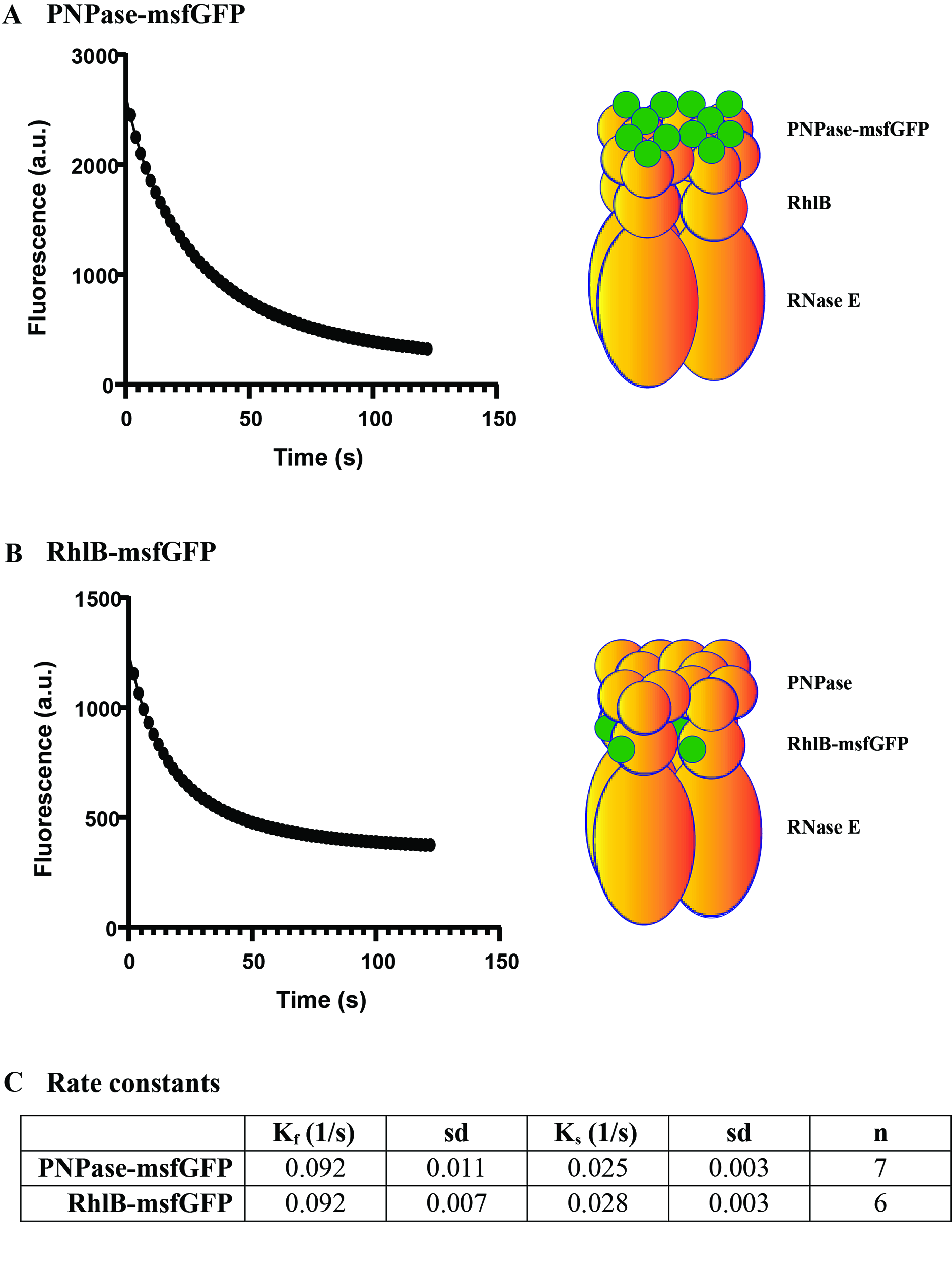

Supplement: FIG S7 [file mbio.01932-21-sf007.tif]

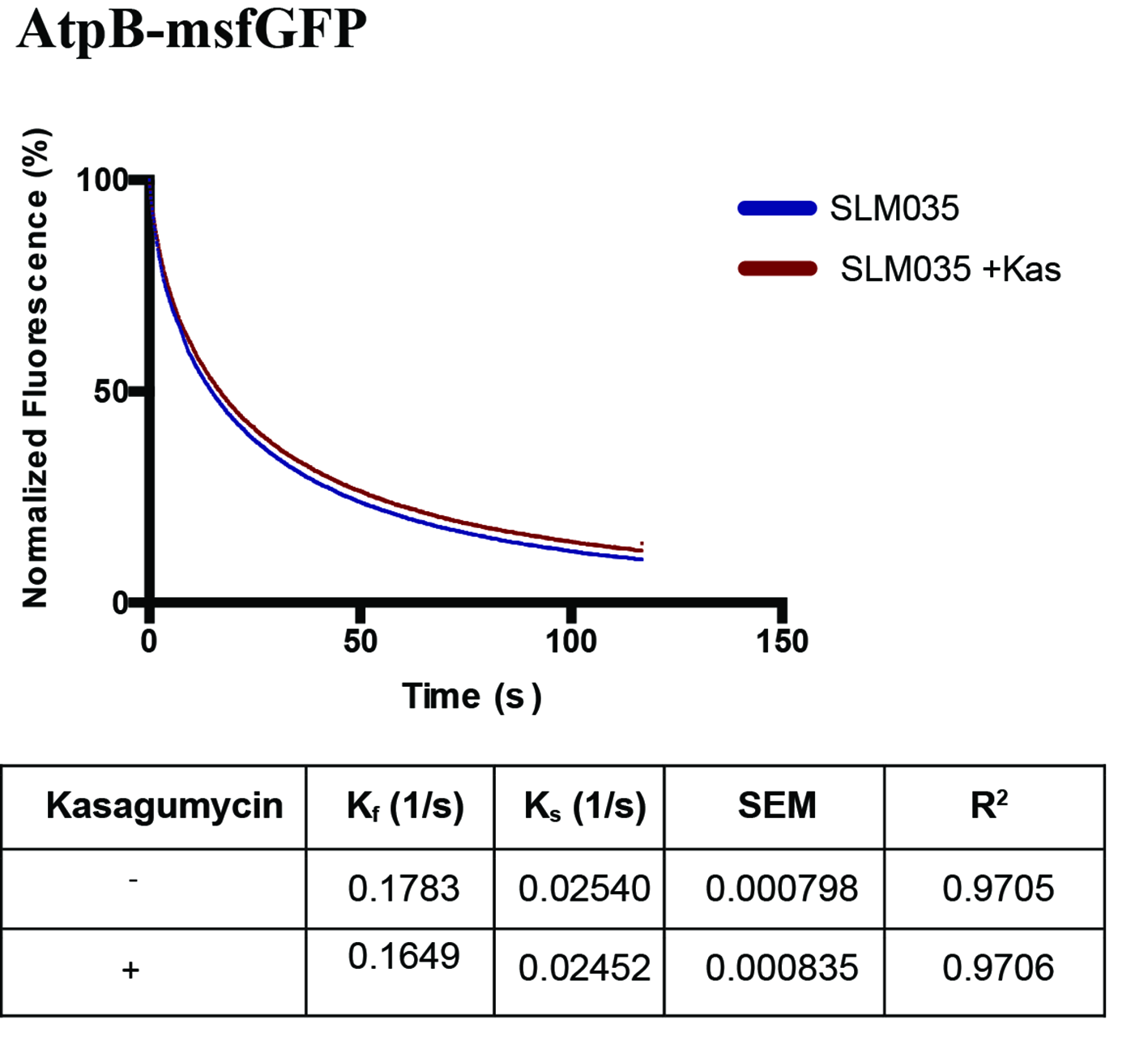

Supplement: FIG S8 [file mbio.01932-21-sf008.tif]

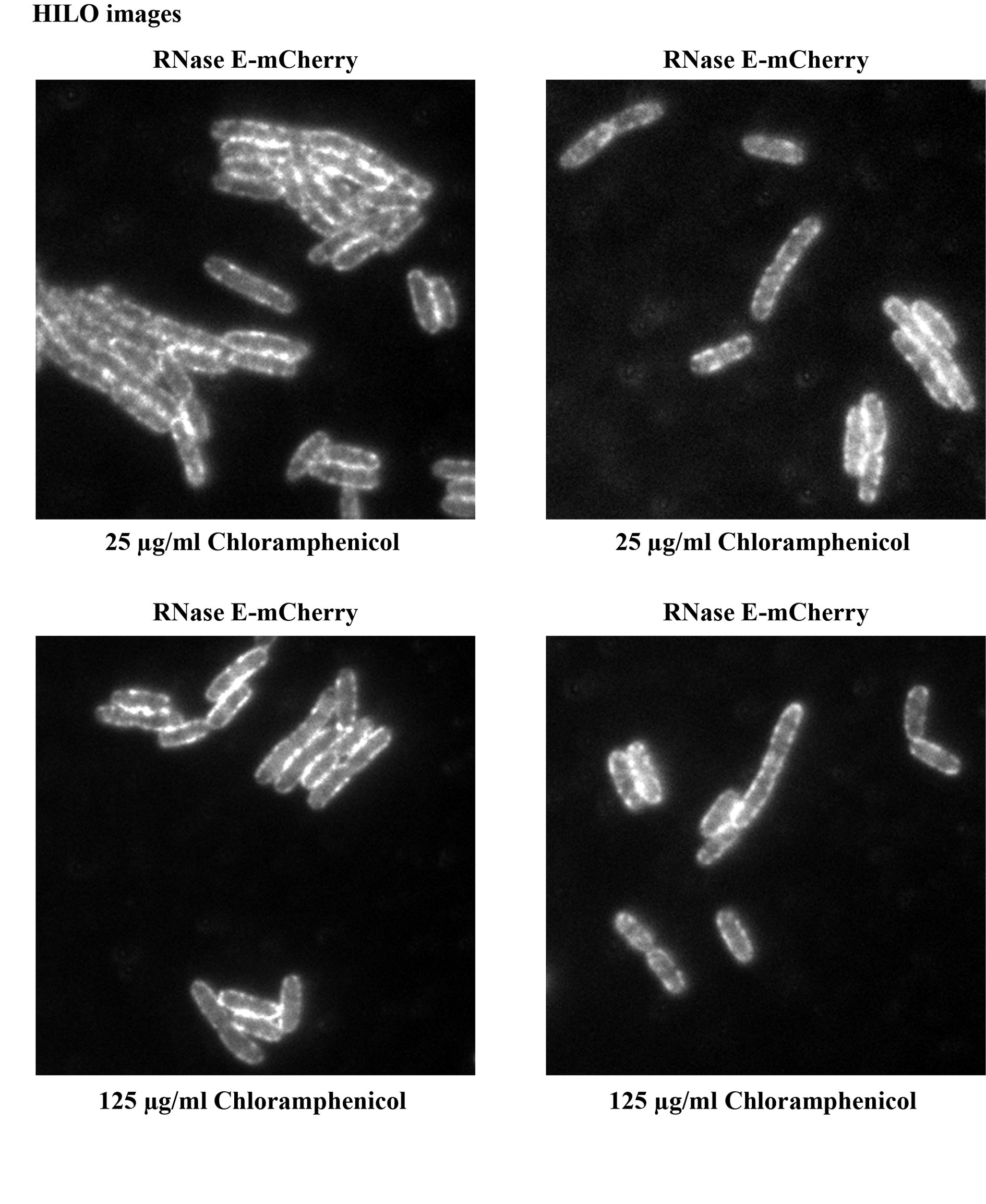

Supplement: FIG S9 [file mbio.01932-21-sf009.tif]
