# Supplementary material for: Polyribosome-Dependent Clustering of Membrane-Anchored RNA Degradosomes To Form Sites of mRNA Degradation in Escherichia coli
Source: mBio. 2021 Sep 7;12(5):e01932-21. doi: 10.1128/mBio.01932-21 (PMC8546579; doi:10.1128/mBio.01932-21)
Supplement: TEXT S1 [file mbio.01932-21-s0001.docx]

**Supplementary Materials and methods**

**Cell imaging growth conditions.** Overnight cultures were diluted to OD_600_ of 0.01 in LB and grown at 37°C with vigorous shaking. After growing to mid logarithmic phase, 1-2 µl were mounted on glass microscope slides with 1.2% (w/v) agarose pads. Drugs were added to shaking cultures 30 min before immobilization on agarose pads: rifampicin 150 µg/ml; kasugamycin 1 mg/ml, chloramphenicol 25 µg/ml and 125 µg/ml. Images were collected immediately at room temperature by epifluorescence and TIRF microscopy.

**Wide field epifluorescence.** For the microscopic analysis of RNA degradosome localization and dynamics, a Nikon Eclipse TI-E/B microscope was used. The microscope is equipped with a phase contrast objective (CFI Plan Fluor DLL 100X oil NA1.3) and Semrock filters sets for GFP (FITC; Ex: 482BP35; DM: 506; Em: 536BP40) and mCherry (Ex: 562BP40; DM: 593; Ex: 641BP75) with exposure times of 0.2 and 1 s respectively. Images were captured and edited using Nis-Elements AR software (Nikon).

**TIRFm imaging**. Images and time-lapse TIRFm videos were taken with an inverted microscope (Nikon Ti-E) equipped with a 100x Nikon immersion oil objective (Apo TIRF, numerical aperture (NA) 1.49) and diode-pumped solid-state lasers (Cobolt Calypso, 40 mW, 488 nm and Cobolt Jive, 50 mW, 561 nm). The exposure time was 200 ms for GFP fusions and ranged between 500 ms to 1s for mCherry fusions. Images were acquired with autofocus (PFS) and with an EMCCD (Electron-Multiplying Charge-Coupled Device) camera (iXON3 Ultra 888, Andor) at maximum gain set to 300, attached to a 1.5x magnification lens and with a final pixel size of 86 nm. For TIRFm time lapse movies, imaging with continuous illumination was performed using the above-mentioned exposure times. To maintain the same depth of evanescent wave penetration as well as the focus position along the acquisitions, laser beam incidence angles and z-position were adjusted individually to obtain epifluorescence, TIRF or HILO illumination modes. Nis‐Elements AR software (Nikon) was used to control image acquisition and editing. All epifluorescence and TIRFm acquisitions were taken independently and at least in duplicate for each strain and type of experiment.

**Affinity purification of native RNA degradosome.**

Overnight cultures of strains SAJ259 and SAJ260 were diluted 100-fold into three 200 ml LB cultures and grown at 37°C to OD_600_=0.6. Cells were pelleted by centrifugation, suspended in 6 ml of IP buffer 2 and then sonicated on ice with a BioBlock Vibracell for 10 cycles (15 s burst - 30 s pause). Lysates were clarified by centrifugation at 10,0000 g for 1 h and then frozen in liquid nitrogen and stored at -80°C. 60 µl of ANTI-FLAG M2 magnetic beads (SIGMA) were pre-washed three times in 2 ml of IP buffer 2 and then incubated with 6 ml of lysate in a 15 ml Falcon tube for 2 hours at 4°C on a rotating platform. The beads were washed three times (x1 6 ml IP buffer 2, x2 6 ml IP buffer 2 without Tween). The beads were then transferred to a 1.5 ml Eppendorf tube and bound proteins were eluted twice with 60 µl of 1 mg/ml Flag peptide in IP buffer 2 without Tween by rotation at 4°C for 30 minutes. The eluates were frozen in 10 µl aliquots in liquid nitrogen and stored at -80°C.

**Nanoscale photobleaching *in vitro*.** 10 µl of affinity purified RNA degradosome was mixed with a Cy5-RNA oligo (0.5 µM final). 1 µl droplets of this mix were deposited on a 24 x 32 x 0.170 mm cover slip. As a reference, 0.5 µl drops of cells expressing PNPase-msfGFP fixed in ethanol were deposited on the same cover slip, which was then affixed to a microscope slide via an adhesive sealing frame (Gene Frame 125 µl, ABgene). Time-lapse videos were made with a Nikon Eclipse TI-E/B wide field epifluorescence microscope using a phase contrast objective (CFI Plan Fluor DLL 100X oil NA1.3) and Semrock filter for GFP (FITC; Ex: 482BP35; DM: 506; Em: 536BP40). The microscope was focused on the surface of the cover slip using the ethanol-fixed reference cells. Droplets containing purified degradosomes and the Cy5-RNA oligo were centered by visualization with a Semrock Cy5 filter (Ex: 628BP40; DM: 660; Em: 692BP40). Time-lapse videos (150 s) were made with continuous excitation of msfGFP. Images were captured every 2 s (exposure times: 0.3 s, PNPase-msfGFP; 1.8 s, RhlB-msfGFP). Videos were analyzed using ImageJ v.1.38 software (National Institutes of Health). Photobleaching was plotted as two phase exponential decay using no constraints (GraphPad Prism version 9.0). Datapoints correspond to total intensities with no background subtraction.
